# Age-related differences in the effectiveness of neuromuscular training for preventing anterior cruciate ligament injuries in athletes: a systematic review and meta-analysis

**DOI:** 10.3389/fpubh.2026.1801019

**Published:** 2026-05-26

**Authors:** Youwei Yao, Xuesong Niu, Naizhao Shao, Yan Yao

**Affiliations:** 1 School of Strength and Conditioning, Shenyang Sport University, Shenyang, Liaoning, China; 2 School of Electronic and Information Engineering, Jinling Institute of Technology, Nanjing, Jiangsu, China; 3 School of Accounting, Nanjing University of Finance and Economics, Nanjing, Jiangsu, China

**Keywords:** age differences, anterior cruciate ligament, meta-analysis, neuromuscular training, sports injury prevention

## Abstract

**Objective:**

Anterior cruciate ligament (ACL) injuries are common in competitive sports and may lead to long-term functional impairment and substantial career burden. Neuromuscular training (NMT) has been proposed to reduce the risk of ACL injury; however, whether its preventive effectiveness differs by age remains controversial. This systematic review and meta-analysis quantitatively evaluated the protective effects of NMT across different age groups.

**Methods:**

Five databases were systematically searched for studies evaluating the effect of NMT on the incidence of ACL injury. Pooled effects were synthesized using random-effects models and reported as odds ratios (ORs) with 95% confidence intervals (CIs). Age-stratified subgroup analyses and meta-regression (using the study-level mean age as a continuous moderator) were conducted to explore potential age-related differences in effectiveness.

**Results:**

A total of 19 studies met the inclusion criteria and contributed to the primary meta-analysis. The random-effects meta-analysis showed that NMT significantly reduced ACL injury risk (OR = 0.456, 95% CI: 0.331–0.627, *p* < 0.001; *I*^2^ = 14.9%). In age-stratified analyses, the effect was significant in athletes aged <18 years (OR = 0.365, 95% CI: 0.252–0.529) but not in those aged ≥18 years (OR = 0.567, 95% CI: 0.291–1.104); the between-subgroup difference was not statistically significant (*p* = 0.258). Meta-regression indicated a positive association between mean age and effect size (*β* = 0.099, *p* = 0.027), suggesting that the protective effect of NMT attenuated with increasing age. Findings were consistent in female-only studies (OR = 0.490, 95% CI: 0.343–0.699, *p* < 0.001). No significant publication bias was detected (Egger’s test *p* = 0.126).

**Conclusion:**

NMT significantly reduces the risk of ACL injury in athletes. Current evidence suggests that its protective effect may be greater in younger athletes and may attenuate with increasing age. As the available evidence is derived predominantly from female cohorts, further research is needed to clarify whether a similar age-related pattern exists in male athletes.

**Systematic review registration:**

https://www.crd.york.ac.uk/PROSPERO/view/CRD420251218902, identifier: CRD420251218902.

## 1 Introduction

Anterior cruciate ligament (ACL) injury is among the most common and consequential knee injuries in sports medicine, occurring frequently in sports such as soccer, basketball, and volleyball that involve repeated cutting, jumping, and landing maneuvers (1–4). A recent U.S. national claims analysis reported a cumulative incidence of ACL tears of 75.19 per 100,000 person-years between 2010 and 2020 (5). Population-based data from Alberta, Canada, likewise indicate a substantial and increasing surgical burden of ACL reconstruction, particularly among females and younger age groups (6). ACL injury typically entails a prolonged rehabilitation process; even after return to sport, athletes remain at elevated risk of reinjury and early-onset osteoarthritis, which can substantially shorten athletic careers (7). Given the high incidence and serious sequelae of ACL injury, proactive preventive interventions are of major importance (8). From a public health perspective, ACL injuries not only impair athletic performance but also contribute to substantial time loss, healthcare utilization, and socioeconomic costs. In addition, they may increase long-term health risks and reduce continued physical activity participation, making ACL injury prevention a relevant priority not only in elite and professional sport systems, but also in community and grassroots sport settings. Existing evidence suggests that training interventions can reduce ACL loading and injury risk by modifying lower-limb kinematics and kinetics—such as flexion, adduction, and rotational angles and moments—as well as ground reaction forces (GRF) during landing, thereby optimizing landing biomechanics (9–15).

Neuromuscular training (NMT), as a standardizable injury-prevention program that can be implemented across club, national team, and professional league settings, is widely regarded as a core strategy for ACL injury prevention (14, 16–18). The central premise of NMT is to enhance coordinated neuromuscular control of movement, improve movement patterns, and increase dynamic joint stability, thereby strengthening lower-extremity motor control (19). Typical NMT programs are multicomponent and may include lower-limb strength training, balance and proprioceptive training, core stability training, agility training, and jump-landing technique training. Although some program features—particularly the inclusion of strengthening and proximal control elements—may be associated with greater preventive effects, the literature more consistently supports multicomponent programs over single-component interventions for ACL injury prevention (20). Prior evidence indicates that the preventive efficacy of NMT may vary according to program composition. Beyond program content, adherence and implementation fidelity may be important determinants of the real-world effectiveness of NMT programs. Subgroup analyses suggest that exercise programs emphasizing strengthening, proximal control, and multiple training components are associated with greater reductions in ACL injury risk, supporting the use of multicomponent rather than single-component preventive programs (21, 22). In contrast to conventional physical conditioning, which primarily targets physical capacities such as strength, speed, power, or endurance, NMT places greater emphasis on movement technique standardization and optimization of neuromuscular control (23). By correcting maladaptive movement patterns and improving coordination and control across the lower-extremity kinetic chain, NMT may reduce ACL injury risk, particularly for non-contact mechanisms that are considered most amenable to prevention (24, 25).

In injury-prevention research, program effectiveness is typically quantified using both absolute occurrence measures, such as the number of injuries, cumulative incidence, or incidence rates based on athlete-exposures or person-time, and relative effect measures comparing intervention and control groups, such as risk ratios, odds ratios, and incidence rate ratios (26–28). Because exposure volume often differs across teams, seasons, and sports, exposure-based incidence metrics and relative effect estimates are generally more informative than raw injury counts alone for between-group comparisons (26, 28). Accordingly, prior trials and meta-analyses have evaluated NMT effectiveness by examining whether it reduces ACL injury occurrence and/or lowers the relative risk of injury, with some syntheses reporting an approximately 50% relative reduction in ACL injury risk in female athletes (25). In recent years, researchers have proposed that the preventive effectiveness of NMT may vary with age and developmental stage (24, 29). The “critical window” hypothesis posits that adolescence may represent an optimal period for implementing NMT to maximize its protective effects. This hypothesis is supported by biomechanical and epidemiological findings showing that ACL injury incidence rises sharply during adolescence and reaches a peak after mid-adolescence, particularly among females (30–32). Adolescence is considered a period of heightened neuromuscular plasticity, during which training interventions may yield substantial improvements in lower-extremity control and movement patterns, thereby enhancing protection (29, 33). Therefore, initiating NMT early in adolescence may confer greater preventive benefits than implementing it in adulthood. This window-of-opportunity framework may also be particularly relevant to community and grassroots sport, where many athletes first accumulate structured training exposure during adolescence, before progressing to higher-performance pathways.

At the same time, important sex differences exist in ACL injury epidemiology and preventive effects (34, 35). Although males may sustain a higher overall incidence of ACL injury in some sports or populations, females have a higher incidence of non-contact ACL injury, particularly after puberty and among age-matched adolescents (36). This distinction is especially relevant in the present context because non-contact ACL injuries are generally considered more amenable to prevention through interventions such as NMT. After puberty, sex-specific disparities in knee biomechanics and neuromuscular control development may contribute to the higher risk of non-contact ACL injury in females (36, 37). Prior studies further suggest that females may benefit more from NMT; for example, in soccer, the reduction in ACL injury rates among female players has been reported to be greater than that among males (38). Accordingly, sex differences may further influence age-related variations in the protective effects of NMT.

Although previous studies indicate that both age and sex may moderate the effectiveness of NMT for ACL injury prevention, systematic quantitative evidence, particularly at the level of continuous age effects, remains limited. Therefore, the aim of this systematic review and meta-analysis was to determine whether age modifies the effectiveness of neuromuscular training for preventing ACL injury in athletes. Specifically, this study aimed to: (1) quantify the preventive effects of NMT across age groups, with a particular focus on whether preventive effects are greater in younger age groups than in older age groups; (2) examine the trend of diminishing effects with increasing age; (3) determine whether consistent age gradients are observed across sexes; and (4) evaluate the robustness of the findings and potential biases, thereby providing evidence to inform age- and sex-stratified ACL prevention strategies. Collectively, these results may also support the development of stratified implementation pathways for ACL prevention within youth development, elite, and professional sport systems.

## 2 Methods

### 2.1 Registration

This systematic review and meta-analysis was conducted in accordance with the Preferred Reporting Items for Systematic Reviews and Meta-Analyses (PRISMA) guidelines. The study protocol was prospectively registered in the International Prospective Register of Systematic Reviews (PROSPERO) (CRD420251218902).

### 2.2 Literature search strategy

A systematic search was performed in PubMed, Embase, Web of Science, the Cochrane Library, and EBSCO (SPORTDiscus) from database inception to September 30, 2025. The search strategy combined keywords and Medical Subject Headings (MeSH) terms, including but not limited to “anterior cruciate ligament”, “ACL”, “neuromuscular training”, “prevention”, “training”, and “athlete.” The full search strategies for all databases are provided in Supplementary Appendix 1.

### 2.3 Eligibility criteria

Eligibility criteria were prespecified according to the PICOS framework. Participants were athletes engaged in systematic training and competition, with no restrictions on sex, age, competitive level, or sport. Eligible studies evaluated NMT interventions aimed at reducing the risk of ACL injury; interventions were required to include at least two relevant components such as strength training, balance training, jump-landing training, proprioceptive training, and movement control training. Single-component interventions were excluded. Control conditions included usual training, routine warm-up, or no specific preventive intervention. Only prospective controlled designs were included, including randomized controlled trials (RCTs) and prospective cohort studies with a comparison group. The primary outcome was ACL injury incidence, with non-contact ACL injury preferentially extracted when explicitly reported. If a study reported only overall ACL injury incidence and did not distinguish injury mechanism, the overall ACL outcome was retained for pooling. Studies were required to report outcome data sufficient to calculate or extract effect estimates for pooling.

Studies were excluded if they involved non-athlete populations; implemented interventions lacking key NMT components; used no comparator or a non-prospective design; did not report ACL injury or lower-extremity injury outcomes; or provided insufficient data to compute or extract effect estimates that could be pooled. Reviews, case reports, conference abstracts, and studies published as abstracts only were excluded, as were studies with inadequate methodological information.

### 2.4 Data extraction

Two reviewers independently extracted data from all included studies. Discrepancies were resolved by discussion and, when necessary, adjudicated by a third reviewer. Extracted information included first author, publication year, country, and study design. Participant characteristics included sample size, sex composition, age information, sport, and competitive level. Intervention characteristics included training components, session duration, weekly frequency, total intervention period, delivery mode, and whether training was supervised; control conditions and follow-up duration were also recorded.

For outcome data, the number of ACL injury events and the corresponding sample size in each group were preferentially extracted to compute effect estimates and confidence intervals. When studies directly reported poolable effect estimates and their confidence intervals (e.g., odds ratios, risk ratios, or incidence rate ratios), these were also extracted and converted as prespecified before pooling. Lower-extremity injury outcomes were extracted using the same principles when reported. For mixed-sex samples, sex-stratified results were extracted when available; if only overall results were reported, sex composition was recorded for subsequent subgroup or meta-regression analyses. When age information, outcome data, or key methodological details were missing, corresponding authors were contacted via email to obtain additional information.

### 2.5 Study selection

Study selection followed the PRISMA 2020 statement. All retrieved records were imported into EndNote and de-duplicated. Two reviewers independently screened titles and abstracts, followed by full-text assessment, and determined study eligibility according to the inclusion and exclusion criteria. Disagreements were resolved by consensus or adjudication by a third reviewer.

### 2.6 Methodological quality assessment

Methodological quality of included RCTs and prospective studies was assessed using the Physiotherapy Evidence Database (PEDro) scale, which evaluates the rigor of study design and reporting. The PEDro scale comprises 11 items (the first item, “eligibility criteria,” is not scored), with a maximum score of 10, covering random allocation, baseline comparability, blinding, completeness of outcome data, and statistical reporting. Each criterion met was awarded 1 point; higher scores indicate better methodological quality. Because some included studies used prospective cohort designs, inherent limitations in randomization and blinding could lead to lower total scores. Nevertheless, the PEDro scale can reflect meaningful differences in data handling, statistical analysis, and follow-up completeness (39, 40). Therefore, PEDro scores were used for descriptive purposes and interpretation rather than as criteria for study inclusion or exclusion.

### 2.7 Statistical analysis

All primary analyses were performed using Comprehensive Meta-Analysis (CMA), version 3.0. Additional REML-based sensitivity analyses were conducted in Stata. Odds ratios (ORs) with 95% confidence intervals (CIs) were used as the summary effect measure. When original studies reported risk ratios (RRs) or incidence rate ratios (IRRs), these were converted to log odds ratios (log ORs) before pooling. Unless otherwise specified, all tests were two-sided with statistical significance set at *p* < 0.05. To account for within- and between-study variance, the DerSimonian–Laird random-effects model was used for the primary analyses. Because biological maturation indicators (e.g., Tanner stage, maturity offset, or age at peak height velocity) were not reported consistently across the included studies, maturation stage could not be incorporated as a subgroup variable or meta-regression moderator. Therefore, chronological age was used as the only developmental indicator reported with sufficient consistency across studies.

To harmonize age data across studies, all age information was converted to a study-level mean age. Among the 19 included studies, 12 reported mean age, six reported only an age range, and one reported a median with a range. When intervention and control group mean ages and sample sizes were reported separately, study-level mean age was calculated as the sample-size–weighted average of the two groups; when only an overall mean was reported, that value was used. For the study reporting a median and minimum–maximum values with a large sample size, the median was treated as an approximation of the mean based on published recommendations, and then combined using sample-size weighting (41). For studies reporting only age ranges without distributional information, the midpoint of the range was used as an approximation of the mean. The harmonized study-level mean age derived after standardization was used for subsequent age-stratified subgroup analyses and random-effects meta-regression. The study-level mean age for each included study is provided in Supplementary Appendix 2.

#### 2.7.1 Primary and subgroup analyses

The primary analysis pooled ACL injury incidence data from all included studies to estimate the overall effect. Age-stratified subgroup analyses were then performed using two classification schemes: (1) < 18 years versus ≥18 years; and (2) < 18 years, 18–20 years, and >20 years. These cutoffs were selected for pragmatic and comparability reasons to facilitate consistent subgrouping across studies.

#### 2.7.2 Random-effects Meta-regression

To examine whether age moderated the preventive effect of NMT on ACL injury, random-effects meta-regression was conducted with study-level mean age as a continuous predictor and log(OR) as the dependent variable. Four models were specified: (1) Mean-age model, using the reported mean age to test the linear association between age and intervention effect; (2) Centered model, centered at 18 years, where the intercept represents the average effect at age 18 (centered age = mean age − 18); (3) Scaled model, based on the centered model and scaled in 5-year units (scaled age = (mean age − 18)/5), such that *β* reflects the change in effect associated with each 5-year increase in age; and (4) Multivariable model, testing the independent association of age after adjustment for covariates including the proportion of females, sample size, and PEDro score.

#### 2.7.3 Assessment of heterogeneity

Between-study heterogeneity was assessed using Cochran’s *Q* test (*α* = 0.10) and the *I*^2^ statistic. *I*^2^ represents the proportion of total variability attributable to true between-study differences, with values of 25, 50, and 75% indicating low, moderate, and high heterogeneity, respectively. Primary analyses were conducted using the DerSimonian–Laird random-effects model. In addition, prespecified subgroup analyses and random-effects meta-regression were performed to examine potential effect modifiers, such as age, and to explore potential sources of between-study differences in effect estimates.

#### 2.7.4 Sensitivity analyses

Sensitivity analyses were conducted to assess the robustness of the pooled estimates. First, leave-one-out analyses were performed by sequentially omitting one study at a time and recalculating the pooled ORs to determine whether the overall results were disproportionately influenced by any single study. Second, as an additional robustness check, the overall and age-stratified subgroup analyses were repeated using a random-effects restricted maximum likelihood (REML) estimator, whereas the primary analyses were based on the DerSimonian–Laird method. Agreement in the direction and overall interpretation of the findings across these analyses was considered to indicate robustness.

#### 2.7.5 Publication Bias

Publication bias was visually assessed using funnel plots and quantitatively evaluated using Egger’s regression test (performed only when the number of included studies was *k* ≥ 10). When *p* < 0.05 suggested potential publication bias, the Duval and Tweedie trim-and-fill method was used to obtain adjusted estimates. Fail-safe N was also reported to quantify the robustness of the results.

## 3 Results

### 3.1 Study selection

**Figure 1:**
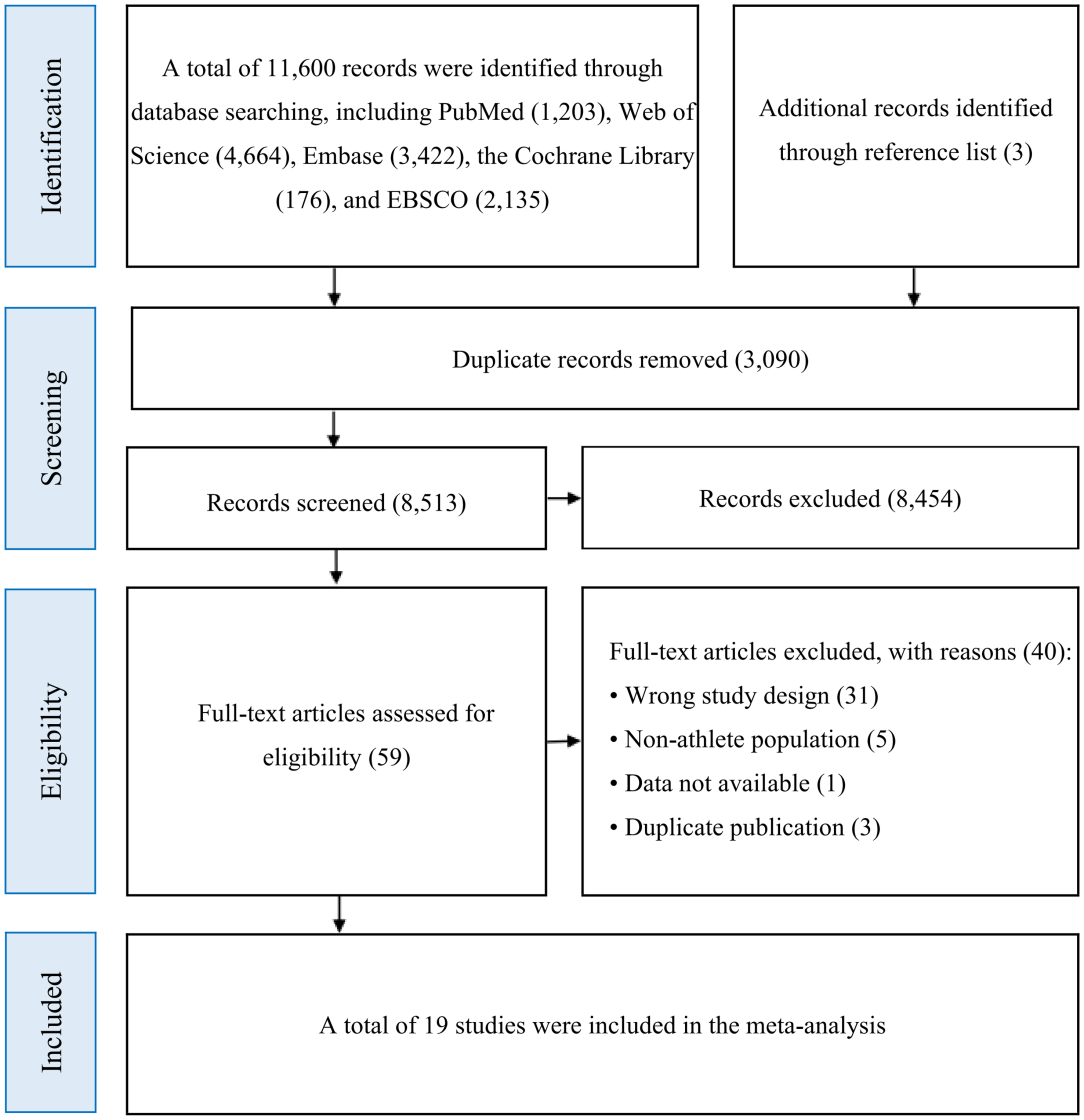
Flow diagram of the literature selection process.

Figure 1 presents the PRISMA flow diagram of study selection. A total of 11,600 records were identified through database searching (PubMed, *n* = 1,203; Web of Science, *n* = 4,664; Embase, *n* = 3,422; Cochrane Library, *n* = 176; EBSCO (SPORTDiscus), *n* = 2,135), and three additional records were identified through reference list screening. After removing duplicates, 8,513 records remained for title and abstract screening, of which 8,454 were excluded. Fifty-nine full-text articles were assessed for eligibility. Of these, 40 were excluded for the following reasons: inappropriate study design (*n* = 31), non-athlete participants (*n* = 5), unavailable data (*n* = 1), and duplicate publications (*n* = 3). Ultimately, 19 studies were included in the meta-analysis.

### 3.2 Characteristics of included studies

Table 1 summarizes the characteristics of the 19 included studies, and more detailed information is provided in Supplementary Appendix 2. The included studies comprised prospective controlled studies or cohort studies (16, 17, 42–45), randomized controlled trials, cluster-randomized controlled trials, or secondary analyses based on randomized cohorts (46–57), as well as one community-based intervention study (58). Most studies included female athletes; only one study involved male soccer players, and two studies included mixed-sex handball samples. The study populations mainly involved athletes participating in soccer, handball, basketball, volleyball, floorball, and sepak takraw, with soccer being the most frequently investigated sport. The interventions were predominantly multicomponent neuromuscular training programs, typically incorporating strength, plyometric, balance, agility, proprioceptive, and technique-related exercises. Overall, the duration of a single training session was generally 15–30 min, although some studies used shorter protocols of 10 min and one study used longer sessions lasting 60–90 min. In several studies, training frequency was typically two to three sessions per week; however, considerable variation was observed across studies, including adjustments between preseason and in-season frequency, implementation before each training session, lower-frequency schedules, and programs conducted four times per week. The duration of the interventions and injury surveillance periods ranged from several weeks to one or more competitive seasons, and some studies also included follow-up across multiple seasons.

**Table 1:** Characteristics of the included studies.

Author (year)	Study design/participants (EG/CG)/Age (years)	Sport	Intervention protocol	Session duration (min/session)	Training frequency (sessions/week)	Intervention duration	ACL injury mechanism	Injury surveillance duration	ACL injury events (EG/CG)
Hewett et al. (1999) (16)	PCS, 366/463, 14–18	Soccer, basketball, volleyball (F)	Flexibility, plyometric, strength	60–90	3	6 weeks	Contact and non-contact	1 season	2/5
Heidt et al. (2000) (42)	PCS, 42/258, 14–18	Soccer (F)	Plyometric, strength, flexibility	NR	3	7 weeks	Mechanism not separated	1 year (2 seasons)	1/8
Myklebust et al. (2003) (43)	PCS, 855/942, 21–22	Handball (F)	Balance, strength, coordination, plyometric, flexibility	15	Pre-season: 3; in-season: 1	2 seasons	Contact and non-contact	3 seasons	23/29
Mandelbaum et al. (2005) (17)	PCS, 1885/3818, 14–18	Soccer (F)	Stretching, strength, plyometric, agility	20	Each training	2 years (2 seasons)	Non-contact ACL only	2 years (2 seasons)	6/67
Olsen et al. (2005) (52)	RCT, 958/879, EG: 16.3 ± 0.6 CG: 16.2 ± 0.6	Handball (F/M)	Warm-up, technique training, balance, plyometric, strength	15–20	Initial phase: each training; thereafter: 1	1 season	Mechanism not separated	1 season	3/10
Petersen et al. (2005) (44)	PCS, 134/142, EG: 19.4 CG: 19.8	Handball (F)	Balance, plyometric	10	Pre-season: 3; in-season: 1	Pre-season: 8 weeks; in-season: NR	Contact and non-contact	1 season	1/5
Pfeiffer et al. (2006) (45)	PCS, 577/862, 14–18	Basketball, soccer, volleyball (F)	Deceleration training, plyometric, agility	20	2	2 seasons	Non-contact ACL only	2 seasons	3/3
Steffen et al. (2008) (55)	RCT, 1073/947, 15.4 ± 0.8	Soccer (F)	Core stability, balance, plyometric, agility, strength	20	Initial phase: each training session for 15 sessions; thereafter: 1	1 season	Mechanism not separated	Preseason and competitive season	4/5
Gilchrist et al. (2008) (49)	RCT, 583/852, 19.88	Soccer (F)	Warm-up, stretching, strength, plyometric, agility	<30	3	12 weeks	Contact and non-contact	1 season	7/18
Pasanen et al. (2008) (53)	RCT, 256/201, 24	Floorball (F)	Running technique training, balance, plyometric, strength	20–30	Intensive phase: 2–3; maintenance phase: 1	6 months (1 season)	Contact and non-contact	6 months (1 season)	6/4
Kiani et al. (2010) (58)	CIS, 777/729, EG: 14.0 [12.7–18.6] CG: 15.0 [13.0–17.6]	Soccer (F)	Warm-up, muscle activation, balance, strength, core stability	20–25	Pre-season: 2; in-season: 1	1 season	Mechanism not separated	Preseason and competitive season	0/5
LaBella et al. (2011) (51)	RCT, 737/755, EG: 16.2 ± 1.5 CG: 16.2 ± 1.1	Soccer, basketball (F)	Strength, balance, plyometric, agility	20	80.4% of practices	13 ± 2.5 weeks	Non-contact ACL only	1 season	2/6
Walden et al. (2012) (56)	RCT, 2479/2085, 14.0 ± 1.2	Soccer (F)	Core stability, balance, knee control	15	2	7 months (1 season)	Contact and non-contact	7 months (1 season)	7/14
Hägglund et al. (2013) (50)	RCT, 2471/2085, 12–17	Soccer (F)	Warm-up, balance, core stability, strength, plyometric	15	2	1 season	Mechanism not separated	1 season	7/14
Achenbach et al. (2017) (46)	RCT, 168/111, EG: 14.9 ± 0.9 CG: 15.1 ± 1.0	Handball (F/M)	Strength, plyometric, jump-landing, proprioceptive training	15	Pre-season: 2–3; competitive season: 1	1 season	Mechanism not separated	1 season	1/2
Silvers-Granelli et al. (2017) (54)	RCT, 675/850, EG: 20.40 ± 1.66 CG: 20.68 ± 1.46	Soccer (M)	Strength, agility, proprioceptive training, plyometric	15–20	2–3	1 season	Contact and non-contact	1 season	3/16
Barber Foss et al. (2018) (47)	RCT, 259/215, 14.0 ± 1.7	Basketball, soccer, volleyball (F)	Plyometric, balance, core stability, strength	Pre-season: 20–25; in-season: 10–15	Pre-season: 3; in-season: 2	1 season	Mechanism not separated	1 season	1/2
Bonato et al. (2018) (48)	RCT, 86/74, EG: 20 ± 2 CG: 20 ± 1	Basketball (F)	Warm-up, flexibility, strength, plyometric	30	4	1 season	Mechanism not separated	1 season	0/7
Yarsiasat et al. (2019) (57)	RCT, 26/26, EG: 15.50 ± 1.10 CG: 15.19 ± 1.26	Sepak takraw (F)	Warm-up, stretching, strength, plyometric, agility	20	3	8 weeks	Mechanism not separated	6 months	1/3

Age is reported in the order of EG and CG and presented as mean, range, mean ± SD, or median [range], as reported in the original studies. Abbreviations: EG, experimental group; CG, control group; ACL, anterior cruciate ligament; PCS, prospective controlled/cohort study; RCT, randomized controlled trial; CIS, community intervention study; NR, not reported; F, female; M, male. In Hewett et al. (16), male athletes served only as an untrained reference group, whereas the intervention comparison was limited to female athletes.

### 3.3 Methodological quality assessment

A total of 19 studies were included and assessed for methodological quality using the PEDro scale. PEDro scores ranged from 2 to 8, with a mean score of 4.6 and a median of 4 (interquartile range: 3.0–6.5) (Table 2). Five studies were classified as high quality (PEDro score ≥ 7), including Waldén et al. (56), Olsen et al. (52), Steffen et al. (55), Pasanen et al. (53), and Yarsiasat et al. (57). Two studies received the lowest scores (score = 2), namely Hewett et al. (16) and Mandelbaum et al. (17). Regarding individual PEDro items, all studies satisfied item 9 (between-group statistical comparisons) and item 10 (reporting of point estimates and measures of variability). In contrast, items 1 (random allocation) and 2 (allocation concealment) were not fulfilled in several studies. Items 4 (blinding of participants) and 5 (blinding of therapists) showed particularly low compliance, with no study fulfilling either item, reflecting practical constraints inherent to exercise-based intervention studies. Item 6 (blinding of outcome assessors) was reported in only a subset of studies and was less consistently met than items related to statistical reporting (items 9–10).

**Table 2:** PEDro scale scores of the included studies.

Author/Year	Total score	1	2	3	4	5	6	7	8	9	10
Hewett et al. (1999) (16)	2							√		√	
Heidt et al. (2000) (42)	4	√					√	√		√	
Myklebust et al. (2003) (43)	4							√	√	√	√
Mandelbaum et al. (2005) (17)	2									√	√
Olsen et al. (2005) (52)	7	√		√			√	√	√	√	√
Petersen et al. (2005) (44)	3			√						√	√
Pfeiffer et al. (2006) (45)	3							√		√	√
Steffen et al. (2008) (55)	7	√		√			√	√	√	√	√
Gilchrist et al. (2008) (49)	3	√		√						√	
Pasanen et al. (2008) (53)	7	√		√			√	√	√	√	√
Kiani et al. (2010) (58)	4			√				√		√	√
LaBella et al. (2011) (51)	4	√						√		√	√
Walden et al. (2012) (56)	8	√	√	√			√	√	√	√	√
Hägglund et al. (2013) (50)	3	√								√	√
Achenbach et al. (2017) (46)	5	√		√				√		√	√
Silvers-Granelli et al. (2017) (54)	4	√		√						√	√
Barber Foss et al. (2018) (47)	4	√						√		√	√
Bonato et al. (2018) (48)	6	√		√				√	√	√	√
Yarsiasat et al. (2019) (57)	7	√		√			√	√	√	√	√

The PEDro scale consists of 11 methodological quality criteria; in this study, 10 items were scored, excluding Item 1 (eligibility criteria), resulting in a total score ranging from 0 to 10. The PEDro items were defined as follows: (1) random allocation; (2) allocation concealment; (3) baseline comparability; (4) blinding of participants; (5) blinding of therapists; (6) blinding of assessors; (7) adequate follow-up (≥85% of participants); (8) intention-to-treat analysis; (9) between-group statistical comparisons; and (10) reporting of point estimates and measures of variability.

### 3.4 Results of the Meta-analysis

#### 3.4.1 Overall analysis

**Figure 2:**
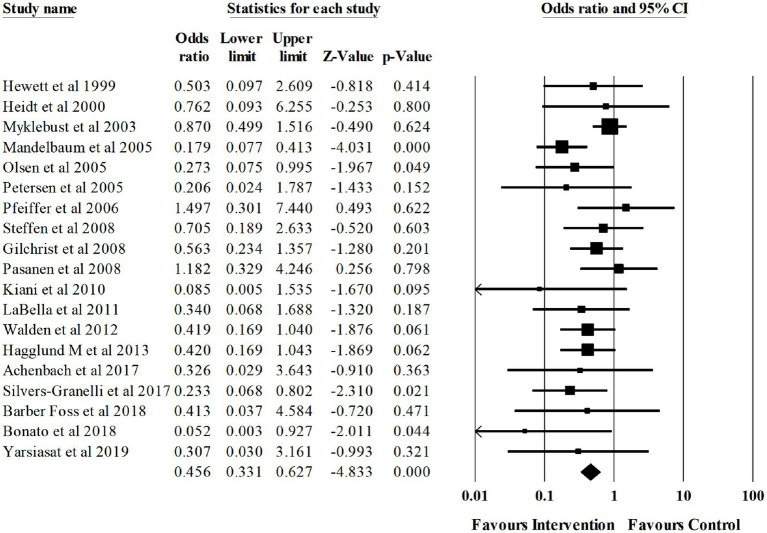
Forest plot of the effect of neuromuscular training on ACL injury risk.

As shown in Figure 2, the random-effects model based on 19 studies indicated that neuromuscular training (NMT) significantly reduced the risk of ACL injury (OR = 0.456, 95% CI: 0.331–0.627, *p* < 0.001). Between-study heterogeneity was low (*I*^2^ = 14.9%). Heterogeneity-related results are provided in Supplementary Appendix 3.

**Figure 3:**
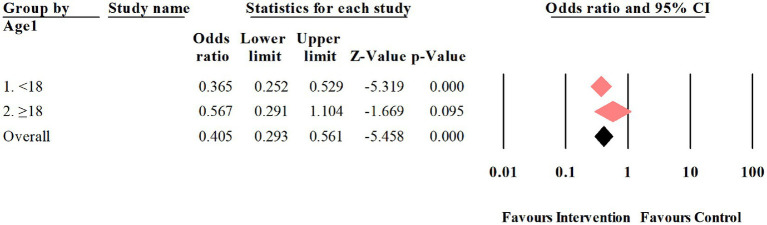
Age-stratified effects of neuromuscular training on ACL injury risk (<18 vs. ≥ 18).

Studies were stratified by study-level mean age into a < 18 years group and a ≥ 18 years group (Figure 3). In the subgroup analysis, neuromuscular training (NMT) was associated with a reduced risk of ACL injury overall (pooled OR = 0.405, 95% CI: 0.293–0.561, *p* < 0.001). A significant protective effect was observed in athletes aged <18 years (OR = 0.365, 95% CI: 0.252–0.529, *p* < 0.001), whereas no significant association was found in athletes aged ≥18 years (OR = 0.567, 95% CI: 0.291–1.104, *p* = 0.095). The between-subgroup difference was not statistically significant (*p* for subgroup difference = 0.258). Overall heterogeneity was low (*I*^2^ = 14.9%).

**Figure 4:**
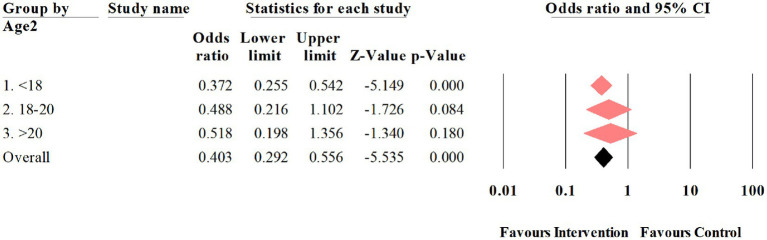
Age-stage–specific effects of neuromuscular training on ACL injury risk (<18 vs. 18–20 vs. >20).

Studies were further categorized into three age groups based on study-level mean age: <18 years, 18–20 years, and >20 years (Figure 4). Using a mixed-effects model, the overall pooled effect was significant (OR = 0.403, 95% CI: 0.292–0.556, *p* < 0.001). In subgroup analyses, a significant protective effect was observed in the <18 years group (OR = 0.372, 95% CI: 0.255–0.542, *p* < 0.001), whereas the effects were not statistically significant in the 18–20 years group (OR = 0.488, 95% CI: 0.216–1.102, *p* = 0.084) or the >20 years group (OR = 0.518, 95% CI: 0.198–1.356, *p* = 0.180). The between-subgroup difference was not statistically significant (p for subgroup difference = 0.721). Overall heterogeneity remained low (*I*^2^ = 14.9%).

#### 3.4.2 Female-only analysis

Because most included studies enrolled female athletes, only one study focused exclusively on males and two studies included mixed-sex samples, which precluded meaningful age-stratified subgroup analyses for male athletes. Therefore, the overall age-stratified findings primarily reflect patterns among female athletes. Using the same approach as described above, we repeated the age-stratified subgroup analyses restricted to female-only studies to assess the robustness of the conclusions.

**Figure 5:**
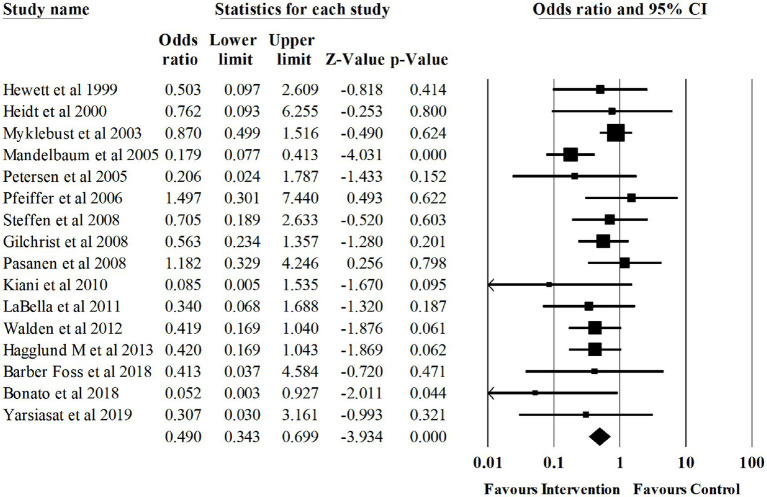
Forest plot of the effect of neuromuscular training on ACL injury risk in female athletes.

As shown in Figure 5, 16 studies conducted in female athletes were included. The random-effects model indicated that neuromuscular training (NMT) was associated with a significantly reduced risk of ACL injury (OR = 0.490, 95% CI: 0.343–0.699, *p* < 0.001). Between-study heterogeneity was low (*I*^2^ = 20.1%).

**Figure 6:**
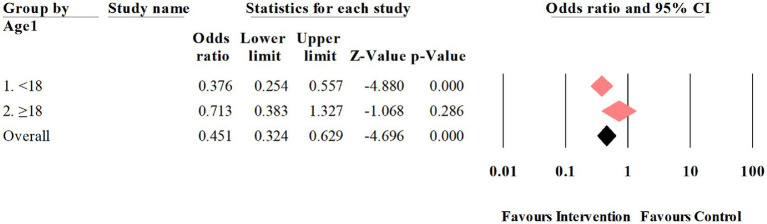
Age-stratified effects of neuromuscular training on ACL injury risk in female athletes (<18 vs. ≥ 18).

The 16 female-only studies were stratified by age into a < 18 years group and a ≥ 18 years group (Figure 6). Using a mixed-effects model, a significant protective effect of neuromuscular training (NMT) was observed in athletes aged <18 years (OR = 0.376, 95% CI: 0.254–0.557, *p* < 0.001), whereas no significant association was found in athletes aged ≥18 years (OR = 0.713, 95% CI: 0.383–1.327, *p* = 0.286). The between-subgroup difference did not reach statistical significance (p for subgroup difference = 0.089). The overall pooled effect across female-only studies remained significant (OR = 0.451, 95% CI: 0.324–0.629, *p* < 0.001), with low heterogeneity (*I*^2^ = 20.1%).

**Figure 7:**
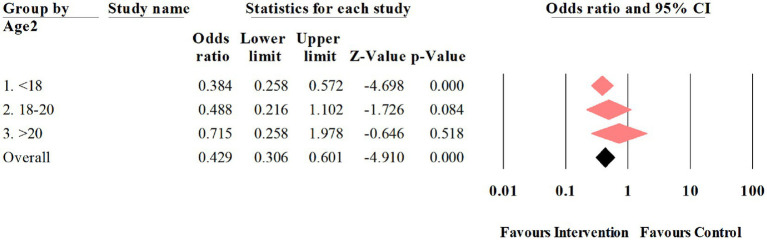
Stage-specific age effects of neuromuscular training on ACL injury risk in female athletes (<18 vs. 18–20 vs. >20).

The 16 female-only studies were further categorized into three age groups based on study-level mean age: <18 years, 18–20 years, and >20 years (Figure 7). Using a mixed-effects model, the overall pooled effect was significant (OR = 0.429, 95% CI: 0.306–0.601, *p* < 0.001). In subgroup analyses, a significant protective effect was observed in the <18 years group (OR = 0.384, 95% CI: 0.258–0.572, *p* < 0.001), whereas the effects were not statistically significant in the 18–20 years group (OR = 0.488, 95% CI: 0.216–1.102, *p* = 0.084) or the >20 years group (OR = 0.715, 95% CI: 0.258–1.978, *p* = 0.518). The between-subgroup difference was not statistically significant (p for subgroup difference = 0.507). Overall heterogeneity remained low (*I*^2^ = 20.1%).

#### 3.4.3 Meta-regression

Random-effects meta-regression analyses were performed using log odds ratios [log (OR)] to examine whether age moderated the preventive effect of neuromuscular training (NMT) on ACL injury risk (Supplementary Appendix 3). In univariable meta-regression of the overall sample, mean age was positively associated with the effect estimate, indicating attenuation of the protective effect with increasing age. Specifically, in the mean-age model (AgeMean), the regression coefficient was *β* = 0.099 (95% CI: 0.011–0.187, *p* = 0.027). Results were unchanged after centering age at 18 years (AgeCentered18: *β* = 0.099, 95% CI: 0.011–0.187, *p* = 0.027). When age was rescaled per 5-year increment (AgePer5), the association remained significant (*β* = 0.496, 95% CI: 0.056–0.935, *p* = 0.027). A positive coefficient indicates that higher mean age is associated with a larger OR (i.e., weaker protective effects of NMT). In the multivariable model, after adjusting for sample size and PEDro score, the age coefficient remained positive but was no longer statistically significant (AgeMean: *β* = 0.081, 95% CI: −0.027 to 0.190; *p* = 0.143).

Female-only studies showed a similarly consistent positive age gradient. In the mean-age model, AgeMean yielded *β* = 0.109 (95% CI: 0.018–0.199, *p* = 0.019). Results were identical in the model centered at 18 years (AgeCentered18: *β* = 0.109, 95% CI: 0.018–0.199, *p* = 0.019). When rescaled per 5-year increment, the association remained significant (AgePer5: *β* = 0.543, 95% CI: 0.090–0.995, *p* = 0.019). Overall, point estimates for the age coefficient were larger and *p* values were smaller in the female-only analyses than in the overall sample across all parameterizations, suggesting a more pronounced age-related gradient in females.

#### 3.4.4 Sensitivity analyses

Leave-one-out sensitivity analyses were conducted for the overall sample (Supplementary Appendix 3). Under the random-effects model, the pooled effect was OR = 0.456 (95% CI: 0.331–0.627, *p* < 0.001). After sequentially removing each study, the pooled OR ranged from 0.394 to 0.539; the lower bounds of the 95% CIs ranged from 0.287 to 0.403 and the upper bounds from 0.541 to 0.722. Across all iterations, the direction of effect was consistent and remained statistically significant (all *p* < 0.001). Sensitivity analyses in the female-only subgroup yielded an overall pooled effect of OR = 0.490 (95% CI: 0.343–0.699, *p* < 0.001). After excluding one study at a time, pooled ORs ranged from 0.422 to 0.597; the lower bounds of the 95% CIs ranged from 0.300 to 0.437 and the upper bounds from 0.596 to 0.815. The protective effect remained consistent in direction and statistically significant in all iterations (all *p* < 0.001).

In addition, we repeated the overall analysis and age-stratified subgroup analyses using a random-effects REML model (Supplementary Appendix 3). In the overall sample, the pooled protective effect of NMT on ACL injury remained statistically significant (OR = 0.452, 95% CI: 0.319–0.638), and the overall pattern of age-stratified results was materially unchanged. The between-subgroup differences were not statistically significant in either the two-category or three-category age analyses (*p* = 0.165 and *p* = 0.710, respectively). In the female-only analyses, the pooled overall effect likewise remained statistically significant (OR = 0.490, 95% CI: 0.337–0.713). The age-stratified results were broadly consistent with the main analyses; the between-subgroup difference reached statistical significance in the two-category age analysis (*p* = 0.024), but not in the three-category age analysis (*p* = 0.076). Taken together, these findings indicate that the main conclusions were robust to the use of an alternative random-effects estimation method.

#### 3.4.5 Publication Bias

Small-study effects and publication bias were assessed using funnel plots, Egger’s regression test, and Begg’s rank correlation test (Supplementary Appendix 3). Visual inspection suggested approximate symmetry of the funnel plots. In the overall analysis, neither Egger’s test (intercept = −0.870, 95% CI: −2.009 to 0.270, *p* = 0.126) nor Begg’s rank correlation test with continuity correction (Kendall’s tau = −0.199, *p* = 0.234) indicated significant asymmetry. Duval and Tweedie’s trim-and-fill analysis identified no missing studies, and the adjusted pooled effect estimate remained unchanged. Similar results were observed in the female-only subgroup, in which neither Egger’s test (intercept = −0.807, 95% CI: −2.087 to 0.472, *p* = 0.197) nor Begg’s rank correlation test with continuity correction (Kendall’s tau = −0.242, *p* = 0.192) suggested significant asymmetry; trim-and-fill analysis likewise did not impute any missing studies.

## 4 Discussion

This systematic review and meta-analysis demonstrated that NMT reduces ACL injury risk in athletes, with stronger preventive effects observed in younger participants (<18 years). From the perspective of the “window of opportunity” hypothesis, initiating NMT during the early stages of neuromuscular development, before ACL injury risk increases substantially, may maximize both its preventive efficacy and long-term benefits (24, 29, 30, 59). Because the current evidence is derived predominantly from female cohorts, this discussion focuses primarily on females. Although NMT has also shown preventive effects in male athletes (54), the limited number of male-only and mixed-sex studies precludes firm conclusions regarding age-related patterns in males.

### 4.1 Potential mechanisms

The age-related analysis in this study was based on study-level age characteristics rather than direct biological measures of pubertal status. Nevertheless, the findings suggest that NMT effectiveness may be influenced by growth and maturation. The observed age-related pattern may reflect the combined influence of biomechanical, neuromuscular, endocrine, and developmental factors (59–61).

With pubertal maturation, female athletes are more likely to develop unfavorable lower-limb movement patterns (61). Compared with prepubertal athletes, postpubertal athletes often exhibit greater knee valgus, reduced knee flexion during landing and cutting, and higher ground reaction forces (GRF) (59, 60). These changes place the knee under greater impact loads in a relatively extended and valgus-aligned position, which is closely linked to increased ACL loading and injury risk (59, 61, 62). In contrast, implementing NMT early may help correct maladaptive movement patterns during rapid skeletal growth, promote safer biomechanics, and potentially reduce later injury risk (32, 33, 63). Rapid adolescent growth is also accompanied by marked changes in body size and segment proportions. If neuromuscular adaptations do not keep pace, coordination and motor control may deteriorate, leading to a “neuromuscular control deficit” and reduced ability to attenuate landing impacts (64). Females may not achieve neuromuscular gains that keep pace with these growth-related changes and may show relative delays in strength and motor control development (62).

As maturation progresses, quadriceps strength relative to body mass may continue to increase while hamstring strength gains lag behind, resulting in an imbalanced quadriceps-to-hamstring profile and a more “quadriceps-dominant” strategy (65–67). This imbalance can increase anterior tibial translation and landing forces in knee extension, thereby elevating ACL loading. Accordingly, NMT programs emphasizing posterior-chain strength and recruitment may help counteract these maturation-related imbalances during the critical adolescent period (68). If the early adolescent window is missed, modifying established movement patterns in adulthood may be more difficult because motor patterns become more entrenched and neural plasticity declines (69–71). Thus, age differences do not necessarily imply that NMT is ineffective in adults; rather, they may reflect reduced plasticity and a smaller benefit from modifying established movement strategies.

Endocrine factors may further contribute to age- and sex-specific patterns. After puberty, increases and cyclical fluctuations in estrogen and relaxin may influence ACL susceptibility through effects on collagen synthesis, ligament laxity, and neuromuscular activation around the knee (72, 73). However, clinical evidence linking ACL injury risk to endogenous hormonal fluctuations across the menstrual cycle or to exogenous hormonal modulation (e.g., oral contraceptive use) remains limited and inconclusive. This uncertainty is further compounded by methodological limitations, including small sample sizes, inconsistent cycle classification, limited biochemical verification, and heterogeneous study designs (74–76).

Beyond biological maturation, the observed age-related pattern may also partly reflect developmental differences in exposure to high-risk sport-specific tasks. During early adolescence, athletes are typically less frequently exposed to high-speed, high-load, and cognitively demanding sport-specific tasks (77, 78). With increasing age, maturation, and progression to higher levels of competition, the physical intensity of sport, game tempo, and perceptual-cognitive demands often increase in parallel, creating more frequent exposure to mechanically disadvantageous positions and greater ACL loading (79–82). Accordingly, the greater preventive benefit observed in younger cohorts may also reflect intervention before substantial cumulative exposure to these high-risk tasks has occurred.

ACL injury risk and the effectiveness of NMT may also be shaped by gendered features of the developmental environment (83). Girls and young women may encounter socially patterned differences in sport participation, training resources, coaching, and support (83–86). These factors may, in turn, influence motor skill acquisition, neuromuscular development, and the timing and quality of preventive training (83, 85).

### 4.2 Comparison with previous evidence

The present findings are broadly consistent with previous evidence and extend the literature through the inclusion of more recent studies and a continuous assessment of age effects. Prior work has identified adolescents—particularly adolescent females—as both a high-risk group for ACL injury and a population that may derive greater benefit from NMT. Prospective intervention studies in adolescent female teams have reported significant reductions in non-contact ACL injuries following NMT implementation (17, 24). Previous meta-analyses have also suggested that athletes younger than 18 years may derive greater benefit than adults, with potential variation across sports (87).

The meta-analysis by Myer et al. (24) focused on female athletes, included 14 prospective controlled trials, and primarily evaluated the preventive effects of NMT through comparisons across age categories. That study found a stronger protective effect in younger age groups and accordingly suggested that earlier implementation of NMT may be more beneficial. In contrast, the present analysis extends the evidence base and methodology in several respects. The literature search was updated through 2025 to include more recently published studies. Sex-based restrictions were not imposed on study inclusion; however, because the available evidence is still derived predominantly from female cohorts, a female-only analysis was additionally performed to examine the robustness of the findings. In addition, eligible interventions were required to include at least two relevant training components, thereby focusing on multicomponent NMT programs. Beyond age-stratified subgroup analyses, meta-regression was also conducted using the study-level mean age as a continuous variable, allowing a more quantitative assessment of the age-related effect. The aim was to evaluate age-related differences in the overall preventive effectiveness of multicomponent NMT, rather than component-specific efficacy or program format. Therefore, component-level comparisons were not performed, and interventions with different training frequencies, session lengths, or intervention durations were not directly compared.

Although the overall evidence suggests that earlier implementation of NMT may yield greater preventive benefits, findings within the younger subgroup were not entirely consistent. These null findings should be interpreted with caution, as they are more likely to reflect methodological issues and differences in intervention implementation than to negate the effectiveness of preventive neuromuscular training itself. Specifically, in one study, the total number of confirmed non-contact ACL injuries was extremely small, with only six cases reported, which may have resulted in insufficient statistical power and unstable effect estimates. In addition, the intervention was delivered twice weekly for approximately 20 min per session and focused primarily on jump-landing and deceleration control; compared with more comprehensive multicomponent NMT programs, the breadth of the training stimulus may therefore have been relatively limited (45). Another study employed a multicomponent preventive program, but the original authors noted that poor compliance may have been an important reason for the absence of a significant effect (55). Compared with younger athletes, the evidence in adults—especially adult females—is less consistent, with several randomized controlled trials showing smaller and less stable effects (48, 49, 53). This pattern is consistent with the age gradient observed here.

### 4.3 Clinical and practical implications

The greater preventive benefit observed in younger cohorts suggests that NMT should ideally be introduced in early adolescence. Neuromuscular training programs implemented before or around the period of maturation, preferably as multicomponent protocols targeting lower-extremity biomechanics and neuromuscular control, such as posterior chain strengthening, proprioceptive training, and core control exercises, may help establish safer movement strategies and reduce ACL injury risk. In contrast, the weaker and less consistent effects observed in older cohorts suggest that programs introduced later may require further optimization of both training content and delivery, for example through an extended maintenance phase and greater emphasis on long-term adherence. More broadly, preventive training programs may benefit from stage-specific strategies, with early implementation in adolescent athletes to establish movement control patterns and continued maintenance and risk-factor management into adulthood. Because NMT can be delivered as a standardized team-based training program, it may be well suited for broad implementation within youth athletic development systems.

### 4.4 Limitations

Several limitations of this study should be acknowledged. First, not all included studies clearly distinguished between contact and non-contact ACL injuries. Although non-contact ACL injury outcomes were preferentially extracted and pooled whenever possible, some pooled estimates were necessarily based on overall ACL injury incidence. Therefore, the present findings should not be interpreted as reflecting exclusively the preventive effects of NMT on non-contact ACL injuries. Second, reporting of prior training history and cumulative lifetime exposure to neuromuscular training or related preventive interventions was inconsistent across studies, which precluded further evaluation of their potential influence on the observed age-related pattern. Thus, the attenuation of the protective effect with increasing age may partly reflect differences in prior training exposure rather than age itself. Third, this study was unable to distinguish biological maturation from chronological age in the analysis. Compared with chronological age, maturation status may more directly reflect neuromuscular development and may therefore be more closely related to responsiveness to NMT. Nevertheless, maturity-related variables were not reported with sufficient consistency or standardization across the included studies to support quantitative synthesis based on biological maturation. Future studies should report indicators such as Tanner stage, maturity offset, or age at peak height velocity more systematically to enable stratified analyses by maturation status.

## 5 Conclusion

This systematic review and meta-analysis indicated that multicomponent neuromuscular training reduces ACL injury risk in athletes. Its protective effect appeared stronger in younger athletes, although the mechanisms underlying this pattern remain unclear. These findings support the routine implementation of NMT as part of team-sport injury prevention. Given that the available evidence is derived predominantly from female cohorts, further studies are needed in male athletes.
