# Supplementary material for: Age-related differences in the effectiveness of neuromuscular training for preventing anterior cruciate ligament injuries in athletes: a systematic review and meta-analysis
Source: Front Public Health. 2026 May 26;14:1801019. doi: 10.3389/fpubh.2026.1801019 (PMC13246426; doi:10.3389/fpubh.2026.1801019)
Supplement: Supplementary file 1 [file Table_1.DOCX]

**Supplementary Appendix 1. Database search strategy**

**Table 1 Search strategy (from inception up to 9 30th, 2025)**

| Headwords  Databases | Search Terms | | |
| --- | --- | --- | --- |
|  | Athletes | Neuromuscular training | Knee/Anterior Cruciate Ligament |
| PubMed  [Title/Abstract] | Athletes [MeSH Terms] OR Athlete  OR Professional Athletes OR Athlete, Professional OR Athletes, Professional OR Professional Athlete OR Elite Athletes OR Athlete, Elite OR Athletes, Elite OR Elite Athlete OR College Athletes OR Athlete, College OR Athletes, College OR College Athlete | neuromuscular OR neuromotor OR NMT OR neuromuscular training OR training OR prevention OR intervention | Knee[MeSH Terms] OR Knee Injuries [MeSH Terms] OR Injuries, Knee OR Injury, Knee OR Knee In  jury OR Anterior Cruciate Ligament [MeSH Terms] OR Cruciate Ligament, Anterior OR Anterior Cruciate Ligaments OR Cruciate Ligaments, Anterior OR Ligament, Anterior Cruciate OR Ligaments, Anterior Cruciate OR Anterior Cruciate Ligament Injuries[MeSH Terms] OR Anterior Cruciate Ligament Injury OR ACL Injuries OR ACL Injury OR Injuries, ACL OR Injury, ACL OR Anterior Cruciate Ligament Tear OR Anterior Cruciate Ligament Tears OR ACL Tears OR ACL Tear OR Tear, ACL OR Tears, ACL OR ACL |
| Cochrane  [Title/Abstract/ keywords] | Athletes[MeSH Terms] OR Athlete OR Professional Athletes OR Athlete, Professional OR Athletes, Professional OR Professional Athlete OR Elite Athletes OR Athlete, Elite OR Athletes, Elite OR Elite Athlete OR College Athletes OR Athlete, College OR Athletes, College OR College Athlete | neuromuscular OR neuromotor OR NMT OR neuromuscular training OR training OR prevention | Knee[MeSH Terms] OR Knee Injuries OR Injuries, Knee OR Injury, Knee OR Knee Injury OR Anterior Cruciate Ligament [MeSH Terms] OR Cruciate Ligament, Anterior OR Anterior Cruciate Ligaments OR Cruciate Ligaments, Anterior OR Ligament, Anterior Cruciate OR Ligaments, Anterior Cruciate OR Anterior Cruciate Ligament Injuries[MeSH Terms] OR Anterior Cruciate Ligament Injury OR ACL Injuries OR ACL Injury OR Injuries, ACL OR Injury, ACL OR Anterior Cruciate Ligament Tear OR Anterior Cruciate Ligament Tears OR ACL Tears OR ACL Tear OR Tear, ACL OR Tears, ACL OR ACL |
| Embase  [Title/Abstract] | Athletes[Emtree term] OR Athlete OR Professional Athletes OR Athlete, Professional OR Athletes, Professional OR Professional Athlete OR Elite Athletes OR Athlete, Elite OR Athletes, Elite OR Elite Athlete OR College Athletes OR Athlete, College OR Athletes, College OR College Athlete | neuromuscular OR neuromotor OR NMT OR neuromuscular training [Emtree term] OR training OR prevention OR intervention | knee[Emtree term] OR knee disease [Emtree term] OR Injuries, Knee OR Injury, Knee OR Knee Injury OR Anterior Cruciate Ligament [Emtree term] OR Cruciate Ligament, Anterior OR Anterior Cruciate Ligaments OR Cruciate Ligaments, Anterior OR Ligaments, Anterior Cruciate OR Ligament, Anterior Cruciate OR Anterior Cruciate Ligament Injury[Emtree term] OR Anterior Cruciate Ligament Injury OR ACL Injuries OR ACL Injury OR Injuries, ACL OR Injury, ACL OR Anterior Cruciate Ligament Tear OR Anterior Cruciate Ligament Tears OR ACL Tears OR ACL Tear OR Tear, ACL OR Tears, ACL OR ACL |
| Web of Science  [Topic] | Athletes OR Athlete OR Professional Athletes OR Athlete, Professional OR Athletes, Professional OR Professional Athlete OR Elite Athletes OR Athlete, Elite OR Athletes, Elite OR Elite Athlete OR College Athletes OR Athlete, College OR Athletes, College OR College Athlete | neuromuscular OR neuromotor OR NMT OR neuromuscular training OR training OR prevention | Knee OR Knee Injuries OR Injuries, Knee OR Injury, Knee OR Knee Injury OR Anterior Cruciate Ligament OR Cruciate Ligament, Anterior OR Anterior Cruciate Ligaments OR Cruciate Ligaments, Anterior OR Ligament, Anterior Cruciate OR Ligaments, Anterior Cruciate OR Anterior Cruciate Ligament Injuries OR Anterior Cruciate Ligament Injury OR ACL Injuries OR ACL Injury OR Injuries, ACL OR Injury, ACL OR Anterior Cruciate Ligament Tear OR Anterior Cruciate Ligament Tears OR ACL Tears OR ACL Tear OR Tear, ACL OR Tears, ACL OR ACL |
| EBSCO  [Abstract] | Athletes OR Athlete OR Professional Athletes OR Athlete, Professional OR Athletes, Professional OR Professional Athlete OR Elite Athletes OR Athlete, Elite OR Athletes, Elite OR Elite Athlete OR College Athletes OR Athlete, College OR Athletes, College OR College Athlete | neuromuscular OR neuromotor OR NMT OR neuromuscular training OR training OR prevention | Knee OR Knee Injuries OR Injuries, Knee OR Injury, Knee OR Knee Injury OR Anterior Cruciate Ligament OR Cruciate Ligament, Anterior OR Anterior Cruciate Ligaments OR Cruciate Ligaments, Anterior OR Ligaments, Anterior Cruciate OR Ligament, Anterior Cruciate OR Anterior Cruciate Ligament Injuries OR Anterior Cruciate Ligament Injury OR ACL Injuries OR ACL Injury OR Injuries, ACL OR Injury, ACL OR Anterior Cruciate Ligament Tear OR Anterior Cruciate Ligament Tears OR ACL Tears OR ACL Tear OR Tear, ACL OR Tears, ACL OR ACL |
