# Supplementary material for: Age-related differences in the effectiveness of neuromuscular training for preventing anterior cruciate ligament injuries in athletes: a systematic review and meta-analysis
Source: Front Public Health. 2026 May 26;14:1801019. doi: 10.3389/fpubh.2026.1801019 (PMC13246426; doi:10.3389/fpubh.2026.1801019)
Supplement: Supplementary file 2 [file Table_2.DOCX]

**Supplementary Appendix 2. Characteristics of included studies**

**Table 1 Characteristics of the included studies**

| **Author (Year)** | **Study Design** | **Evidence Level** | **Sample size** | **Age (years)** | **Study-level mean age (years)** | **Sport** | **Training content** | | **Session duration (min/session)** | **Training frequency**  **(sessions/week)** | **Intervention duration** | **ACL injury mechanism** | **Injury surveillance duration/**  **follow-up duration** | **Number of ACL injuries** |
| --- | --- | --- | --- | --- | --- | --- | --- | --- | --- | --- | --- | --- | --- | --- |
|  |  |  |  |  |  |  | **EG** | **CG** |  |  |  |  |  |  |
| Hewett TE et al., 1999 | Prospective controlled study | Ⅱ | EG: 366  CG: 463 | 14-18 | 16.00 | Soccer, basketball, volleyball (F) | Flexibility, plyometric, strength | Usual training | **60–90** | **3** | 6 weeks | **Contact and non-contact** | **1 season** | EG:2  CG: 5 |
| Heidt RS et al., 2000 | Prospective non-randomized controlled cohort study | II | EG 42  CG 258 | 14-18 | 16.00 | Soccer (F) | Plyometric, strength, flexibility | No training | NR | 3 | 7 weeks | **Mechanism not separated** | **1 year**  **(2 seasons)** | EG:1  CG:8 |
| Myklebust G et al., 2003 | **Prospective non-randomized controlled cohort study (cluster allocation by clubs)** | II | EG:855  CG:942 | 21-22 | 21.50 | Handball (F) | Balance, strength, coordination, plyometric, flexibility | Usual warm-up | 15 | Pre-season: 3; in-season: 1 | 2 seasons | **Contact and non-contact** | **3 seasons** | EG：23  CG：29 |
| Mandelbaum BR et al., 2005 | Prospective non-randomized cohort study (two-season follow-up) | II | EG:1885  CG:3818 | 14–18 | 16.00 | Soccer (F) | Stretching, strength, plyometric, agility | Usual warm-up | 20 | Each training | **2 years**  **(2 seasons)** | **Non-contact ACL only** | **2 years**  **(2 seasons)** | EG:6  CG:67 |
| Olsen OE et al., 2005 | Cluster randomized controlled trial (clubs as the unit of randomization) | I | EG:958  CG:879 | EG：16.3±0.6  CG：16.2±0.6 | 16.25 | Handball (F/M) | Warm-up, technique training, balance, plyometric, strength | Usual training | 15–20 | **Initial phase: each training ; thereafter: 1** | 1 season | **Mechanism not separated** | **1 season** | EG:3  CG:10 |
| Petersen W et al., 2005 | Prospective controlled study (controlled prospective case–control design) | II | EG:134  CG:142 | EG：19.4  CG：19.8 | 19.61 | Handball (F) | Balance, plyometric | Usual training | 10 | Pre-season: 3; in-season: 1 | Pre-season:  8 weeks;  in-season: NR | **Contact and non-contact** | **1 season** | EG:1  CG:5 |
| Pfeiffer RP et al., 2006 | Prospective cohort study | II | EG:577  CG:862 | 14-18 | 16.00 | Basketball, soccer, volleyball (F) | Deceleration training, plyometric, agility | Usual training | 20 | **2** | **2 seasons** | **Non-contact ACL only** | **2 seasons** | EG:3  CG:3 |
| Steffen K et al., 2008 | **Cluster randomized controlled trial** | I | EG:1073 CG:947 | 15.4±0.8 | 15.40 | Soccer (F) | Core stability, balance, plyometric, agility, strength | Usual warm-up and training | 20 | Initial phase:  each training session for 15 sessions; thereafter: 1 | **1 season** | **Mechanism not separated** | **Preseason and competitive season** | EG4  CG:5 |
| Gilchrist J et al., 2008 | Cluster randomized controlled trial (cluster RCT) | I | EG:583  CG:852 | 19.88 | 19.88 | Soccer (F) | Warm-up, stretching, strength, plyometric, agility | Usual warm-up and training | <30 | 3 | 12 weeks | **Contact and non-contact** | **1 season** | EG:7  CG:18 |
| Pasanen K et al., 2008 | Cluster randomized controlled trial (cluster RCT) | Ⅰ | EG:256  CG:201 | 24 | 24.00 | Floorball (F) | Running technique training, balance, plyometric, strength | Usual training | 20–30 | Intensive phase: 2–3; maintenance phase: 1 | 6 months  (1 season) | **Contact and non-contact** | 6 months  (1 season) | EG:6  CG:4 |
| Kiani A et al., 2010 | Community-based intervention trial | II | EG:777  CG:729 | EG:14.0[12.7–18.6]  CG:15.0[13.0–17.6] | 14.48 | Soccer (F) | Warm-up, muscle activation, balance, strength, core stability | Usual warm-up and training | 20–25 | Pre-season: 2;  in-season: 1 | 1 season | **Mechanism not separated** | **Preseason and competitive season** | EG:0  CG: 5 |
| LaBella CR et al., 2011 | **Cluster randomized controlled trial (cluster RCT)** | I | EG:737  CG:755 | EG：16.2 ± 1.5  CG：16.2 ± 1.1 | 16.20 | Soccer, basketball (F) | Strength, balance, plyometric, agility | Usual warm-up | 20 | **80.4% of practices** | **13 ± 2.5** weeks | **Non-contact ACL only** | **1 season** | EG:2  CG:6 |
| Walden M et al., 2012 | Stratified cluster randomized controlled trial | I | EG:2479  CG:2085 | 14.0±1.2 | 14.00 | Soccer (F) | Core stability, balance, knee control | Usual warm-up and training | **15** | **2** | **7 months**  **(1 season)** | **Contact and non-contact** | **7 months**  **(1 season)** | EG:7  CG: 14 |
| Hägglund M et al., 2013 | Secondary analysis of a randomized controlled trial (prospective cohort analysis based on a cluster RCT) | II | EG:2471  CG:2085 | 12-17 | 14.50 | Soccer (F) | Warm-up, balance, core stability, strength, plyometric | Usual training | **15** | 2 | 1 season | **Mechanism not separated** | **1 season** | EG:7  CG:14 |
| Achenbach L et al., 2017 | Cluster randomized controlled trial | I | EG:168  CG:111 | EG: 14.9 ± 0.9  CG: 15.1 ± 1.0 | 14.98 | Handball (F/M) | Strength, plyometric, jump-landing, proprioceptive training | Usual training | **15** | Pre-season: 2–3; competitive season: 1 | 1 season | **Mechanism not separated** | **1 season** | EG:1  CG: 2 |
| Silvers-Granelli HJ et al., 2017 | Prospective cluster randomized controlled trial | I | EG:675  CG:850 | EG：20.40±1.66  CG：20.68±1.46 | 20.56 | Soccer (M) | Strength, agility, proprioceptive training, plyometric | Usual warm-up and training | **15–20** | 2–3 | 1 season | **Contact and non-contact** | **1 season** | EG:3  CG: 16 |
| Barber Foss KD et al., 2018 | Prospective randomized controlled clinical trial (cluster RCT) | I | EG:259  CG:215 | 14.0±1.7 | 14.00 | Basketball, soccer, volleyball (F) | Plyometric, balance, core stability, strength | **Elastic-band resisted running** | **Pre-season: 20–25; in-season: 10–15** | Pre-season: 3;  in-season: 2 | 1 season | **Mechanism not separated** | **1 season** | EG:1  CG:2 |
| Bonato M et al., 2018 | Cluster randomized controlled trial | I | EG:86  CG:74 | EG 20±2 ；  CG 20±1 | 20.00 | Basketball (F) | Warm-up, flexibility, strength, plyometric | Usual training | 30 | 4 | 1 season | **Mechanism not separated** | **1 season** | EG:0  CG:7 |
| Yarsiasat J et al., 2019 | Randomized controlled trial (cluster RCT) | I | EG:26  CG:26 | EG 15.50±1.10  CG 15.19±1.26 | 15.35 | Sepak takraw (F) | Warm-up, stretching, strength, plyometric, agility | Usual warm-up | 20 | 3 | 8 weeks | **Mechanism not separated** | **6 months** | EG:1  CG:3 |

**Notes:** Age is presented in the order of the experimental group (EG) and control group (CG). A single value indicates the mean age; a–b indicates an age range; x ± y indicates mean ± standard deviation; and x [a–b] indicates median [range]. Abbreviations: EG, experimental group; CG, control group; ACL, anterior cruciate ligament; PCS, prospective cohort study; RCT, randomized controlled trial; CIS, community intervention study; F, female; M, male. In Hewett et al. (1999), male athletes were included as an untrained reference group only; the intervention comparison was conducted exclusively in female athletes.
