# Supplementary material for: Age-related differences in the effectiveness of neuromuscular training for preventing anterior cruciate ligament injuries in athletes: a systematic review and meta-analysis
Source: Front Public Health. 2026 May 26;14:1801019. doi: 10.3389/fpubh.2026.1801019 (PMC13246426; doi:10.3389/fpubh.2026.1801019)
Supplement: Supplementary file 3 [file Table_3.DOCX]

****Supplementary Appendix 3. Statistical results**
**1. Effect size and heterogeneity analyses**
**1.1 Overall sample****

**
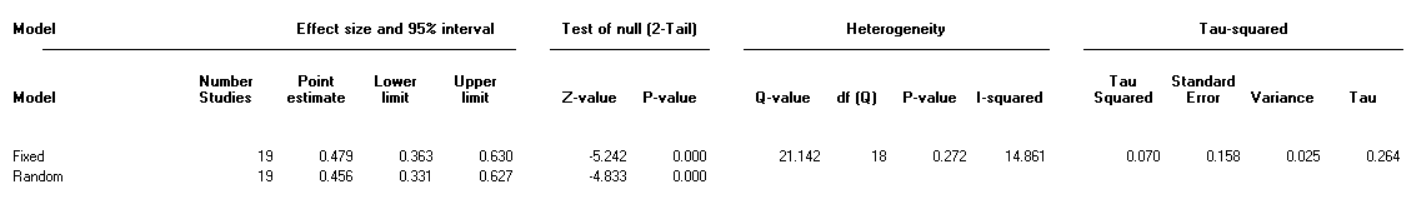
**

Figure 1 Effect size and heterogeneity results


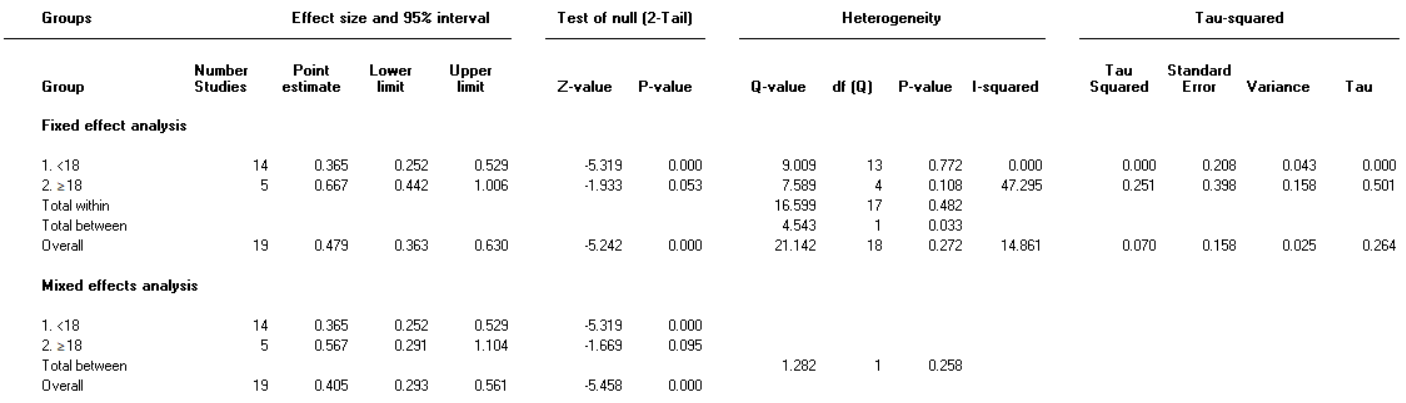


Figure 2 Effect size and heterogeneity results (＜18 vs ≥18)


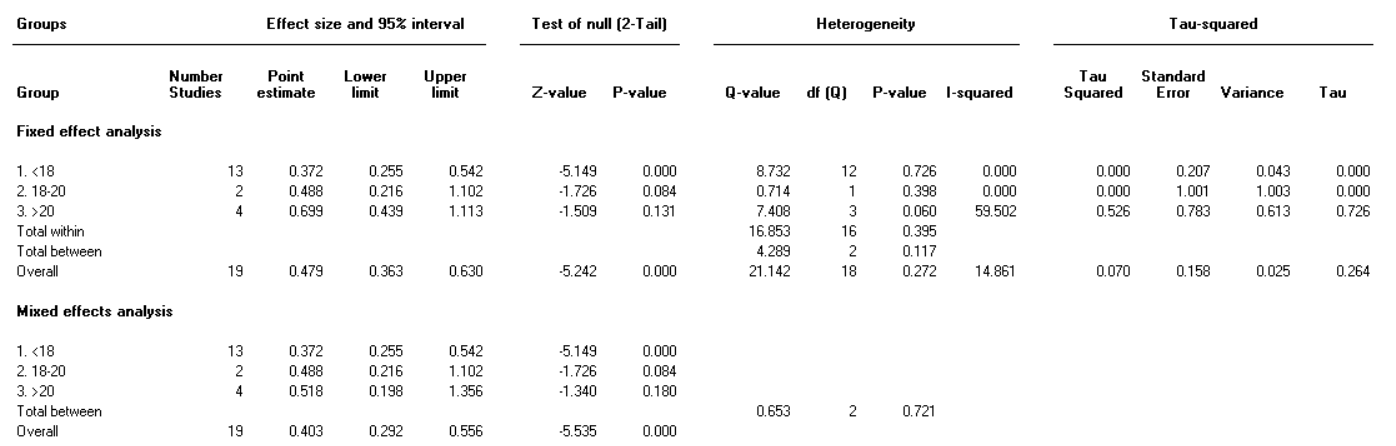


Figure 3 Effect size and heterogeneity results (<18, 18–20, and >20)

**1.2 Female sample**


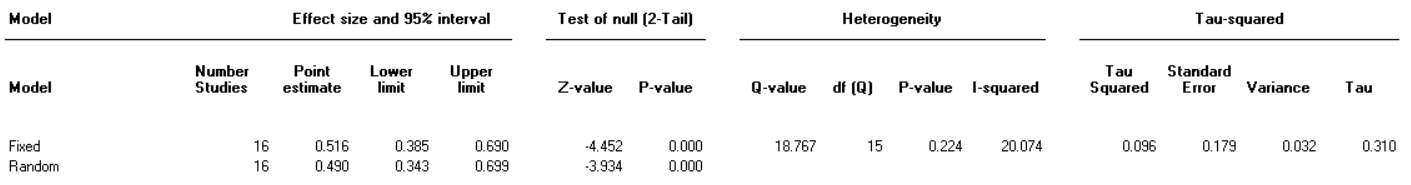


Figure 4 Effect size and heterogeneity results

**
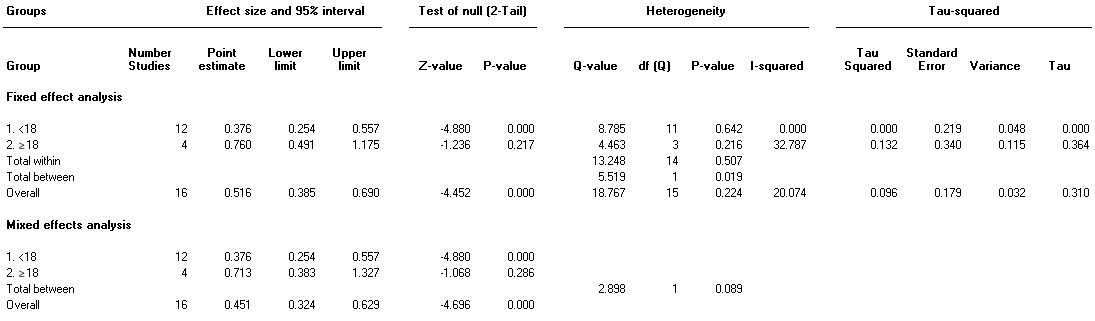
**

Figure 5 Effect size and heterogeneity results (＜18 vs ≥18)

**
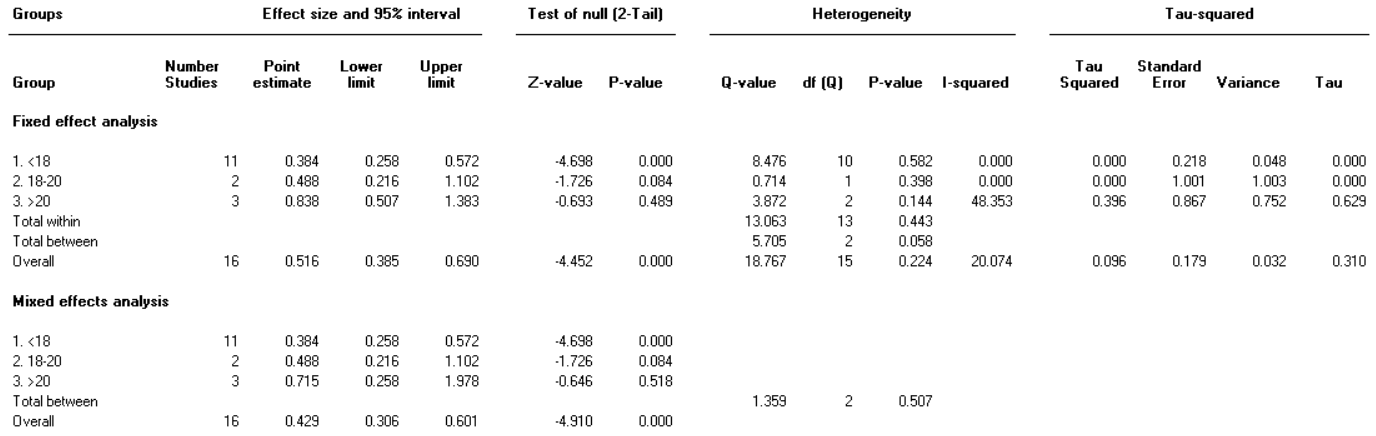
**

Figure 6 Effect size and heterogeneity results (<18, 18–20, and >20)

### ****2 Meta-regression analyses****

#### ****2.1 Overall sample****

**
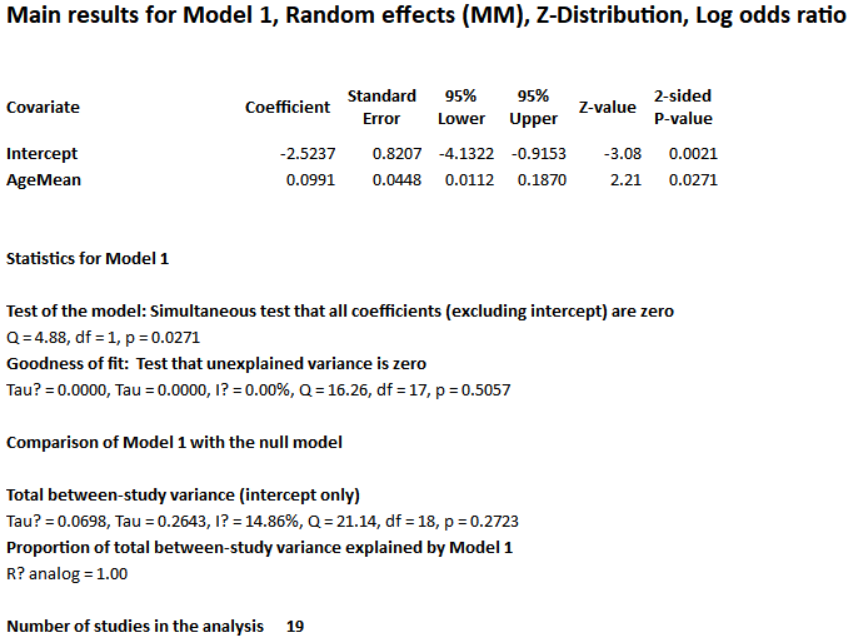
**

Figure 7 Random-effects meta-regression analysis of the association between mean age and intervention effect

Figure 8 Meta-regression bubble plot showing the relationship between mean age and effect size


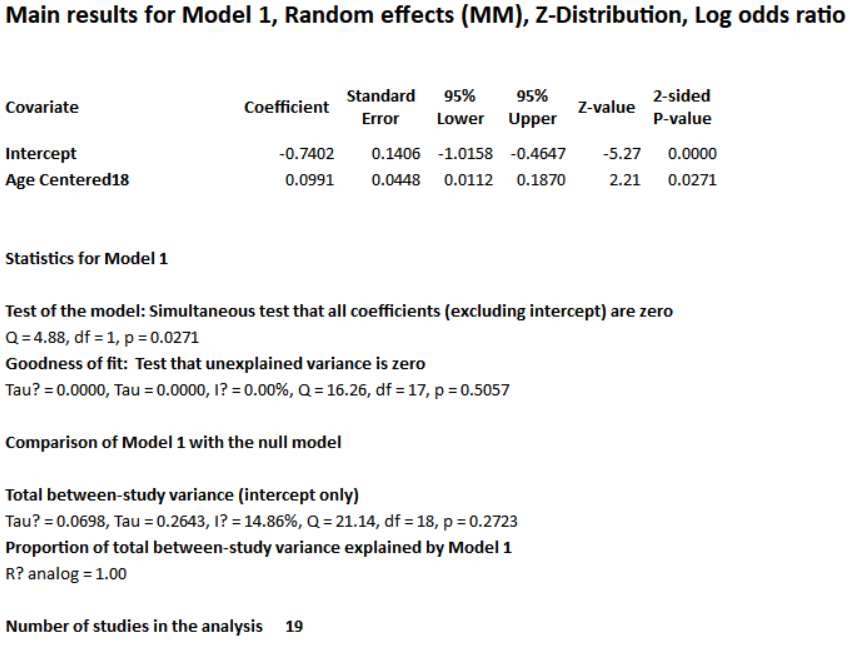


Figure 9 Random-effects meta-regression analysis of the association between mean-centered age and intervention effect

Figure 10 Meta-regression bubble plot showing the relationship between mean-centered age and effect size


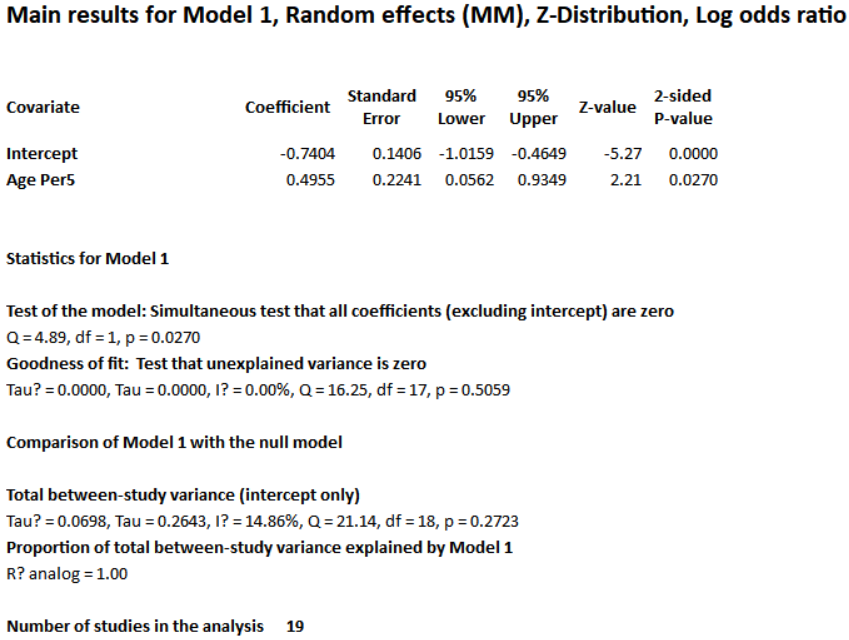


Figure 11 Random-effects meta-regression analysis of the association between standardized age and intervention effect

Figure 12 Meta-regression bubble plot showing the relationship between standardized age and effect size


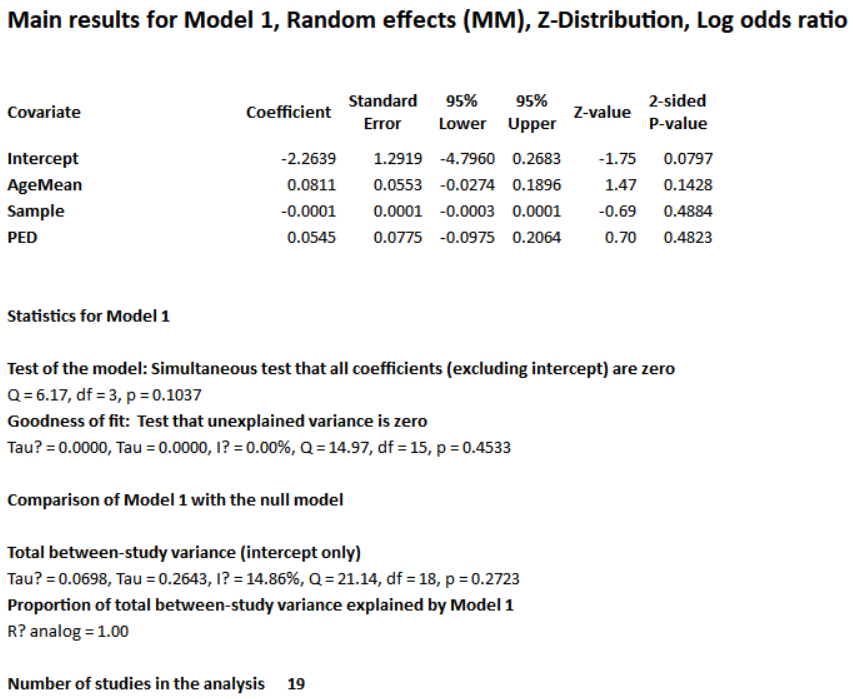


Figure 13 Multivariable random-effects meta-regression analysis

Figure 14 Meta-regression bubble plot showing the relationship between the multivariable model and effect size

**2.2 Female sample**


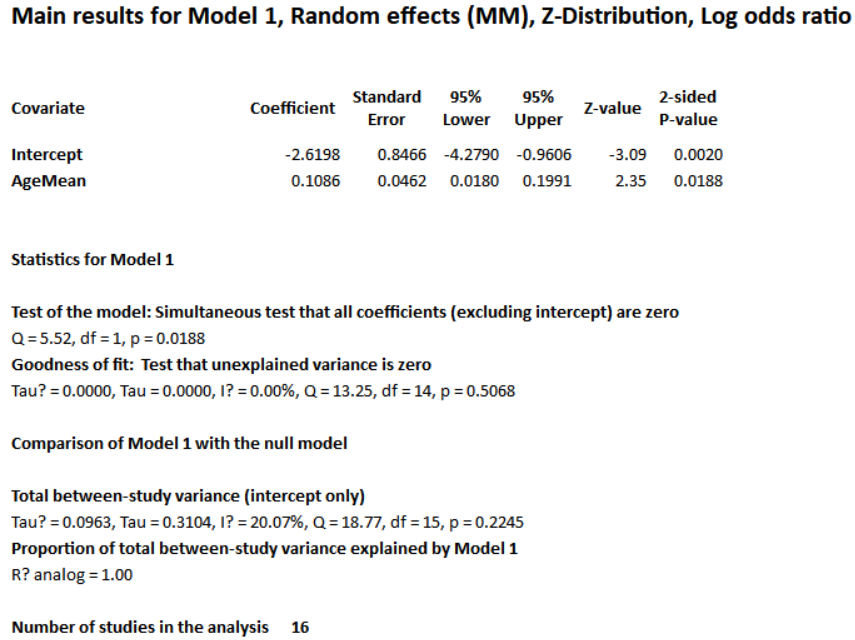


Figure 15 Random-effects meta-regression analysis of the association between mean age and intervention effect

Figure 16 Meta-regression bubble plot showing the relationship between mean age and effect size


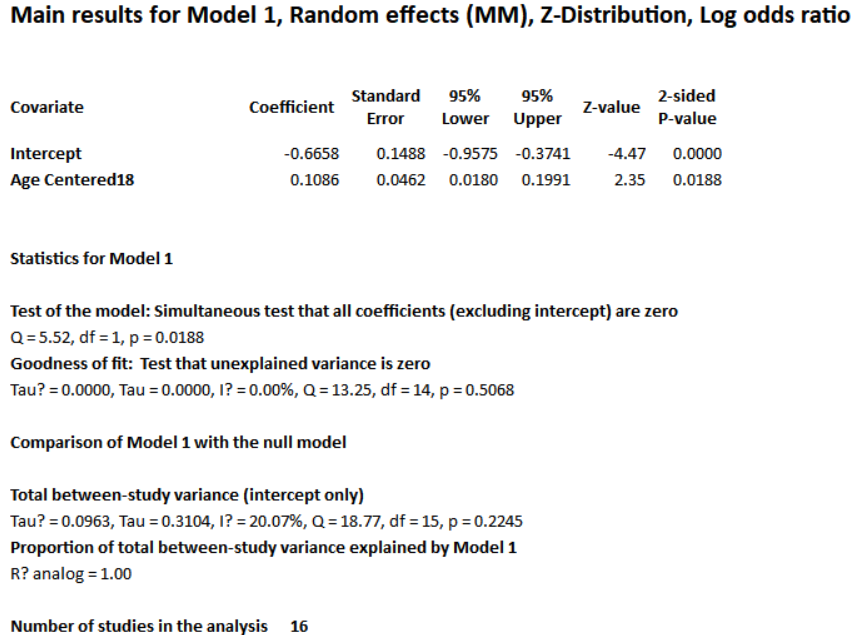


Figure 17 Random-effects meta-regression analysis of the association between mean-centered age and intervention effect

Figure 18 Meta-regression bubble plot showing the relationship between mean-centered age and effect size

**
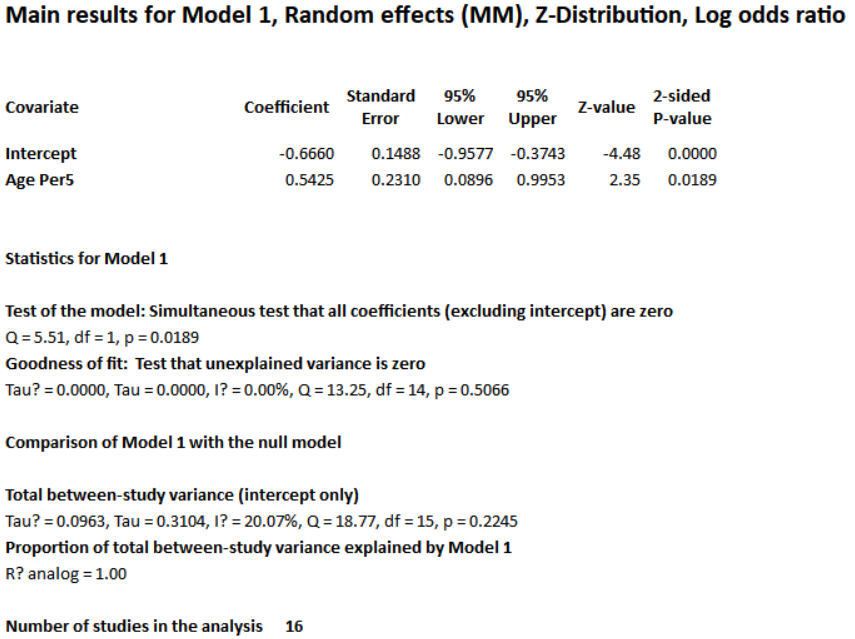
**

Figure 19 Random-effects meta-regression analysis of the association between standardized age and intervention effect

Figure 20 Meta-regression bubble plot showing the relationship between standardized age and effect size

## ****3 Sensitivity analyses****

**3.1 Leave-one-out sensitivity analyses**

### ****3.1.1 Overall sample****

**
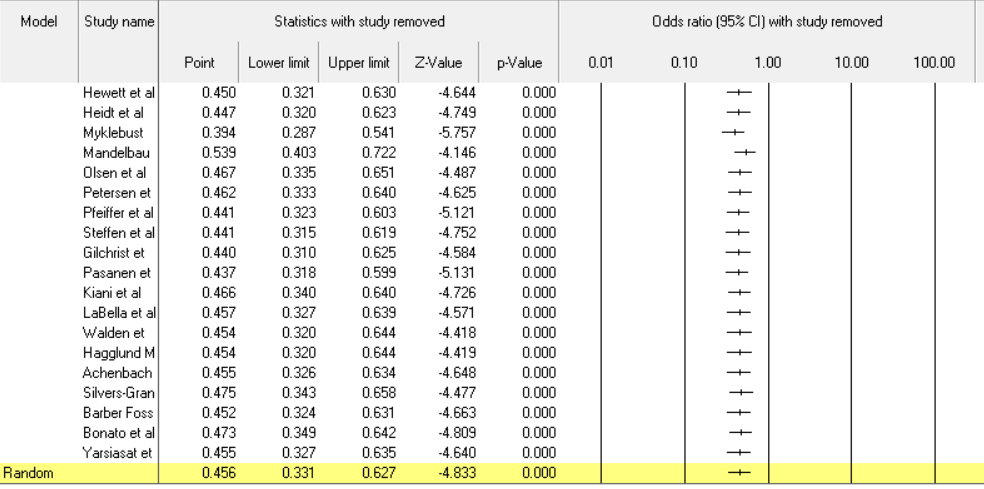
**

Figure 21 Sensitivity analysis using the leave-one-out method

****3.1.2 Female sample****


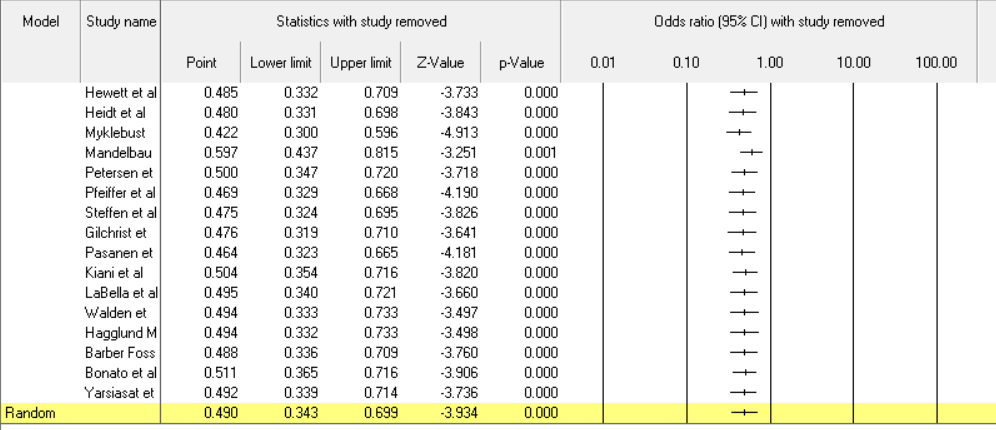


Figure 22 Sensitivity analysis using the leave-one-out method

**3.2 REML sensitivity analysis**

The REML models and corresponding forest plots in this section are presented on the log odds-ratio scale. For ease of interpretation, pooled estimates and their 95% confidence intervals reported in the main text were exponentiated and presented as odds ratios (ORs).

**3.2.1 Overall sample**

**3.2.1.1 Data Results**


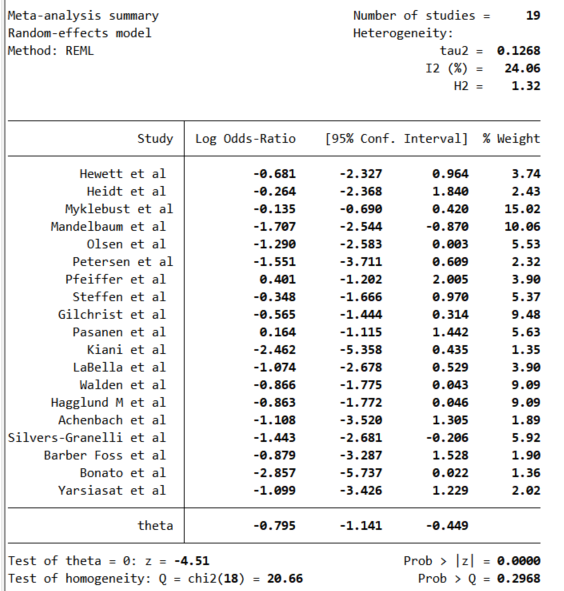


**Figure 23 REML sensitivity analysis results**


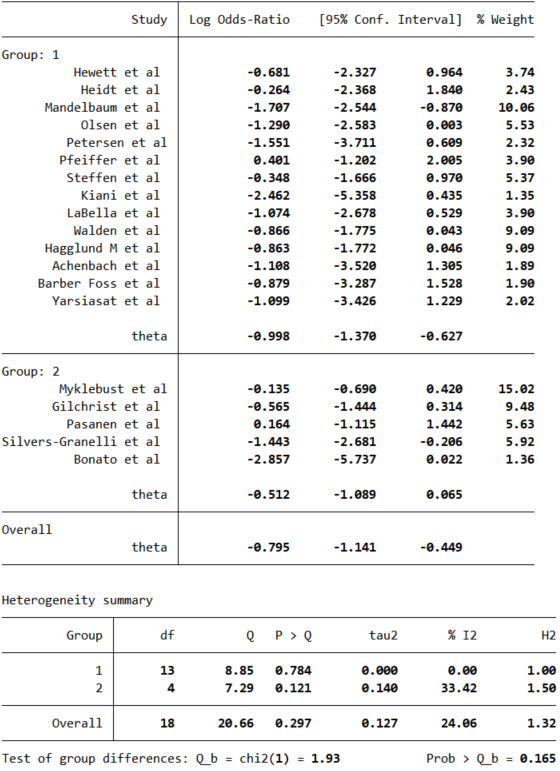


**Figure 24 REML sensitivity analysis results for the dichotomized age subgroup analysis**


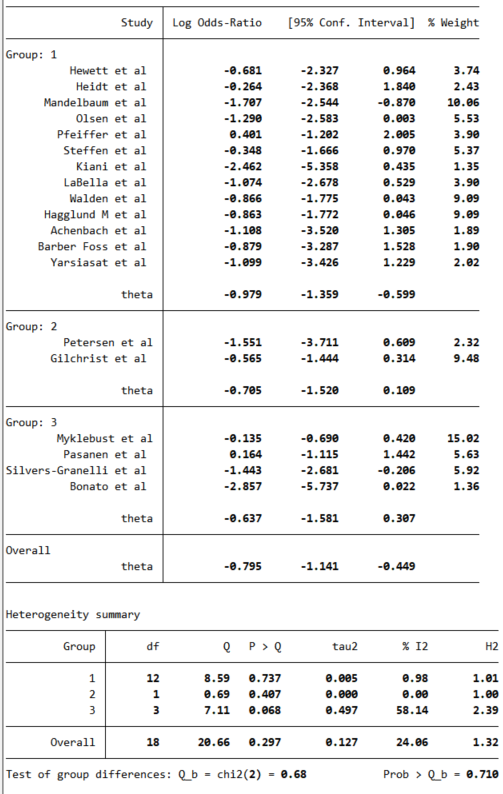


**Figure 25 REML sensitivity analysis results for the three-category age subgroup analysis**

**3.2.1.2 Forest plots**

**
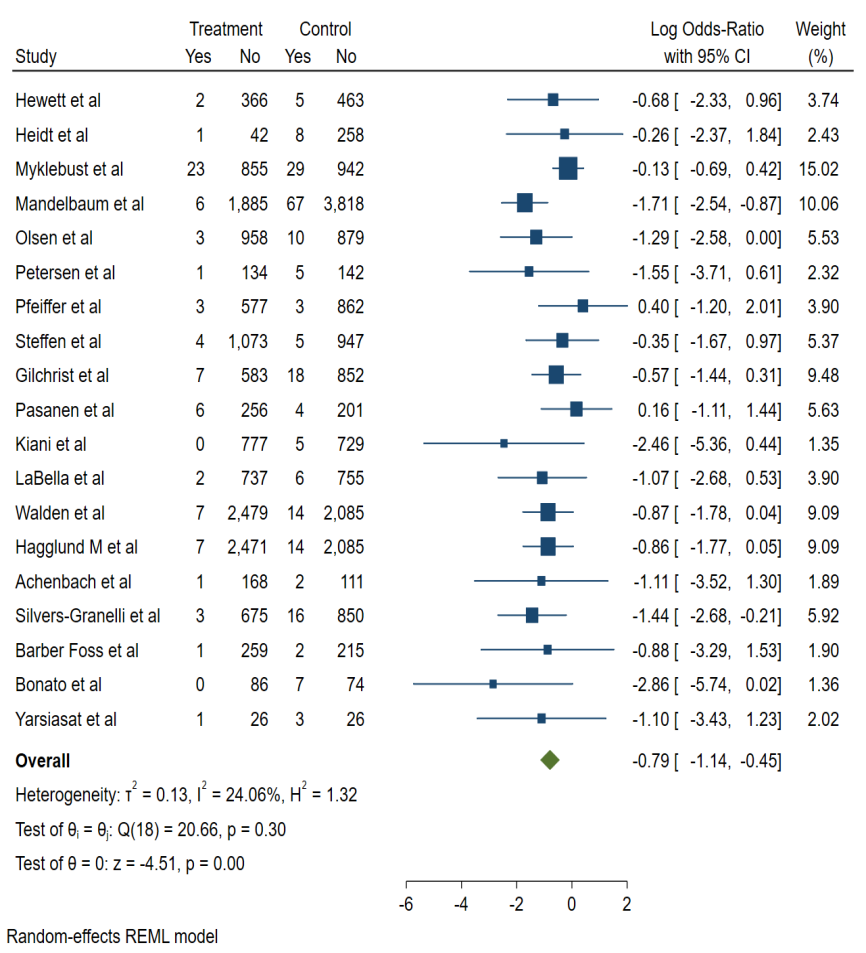
**

**Figure 26 Forest plot for the REML sensitivity analysis**

**
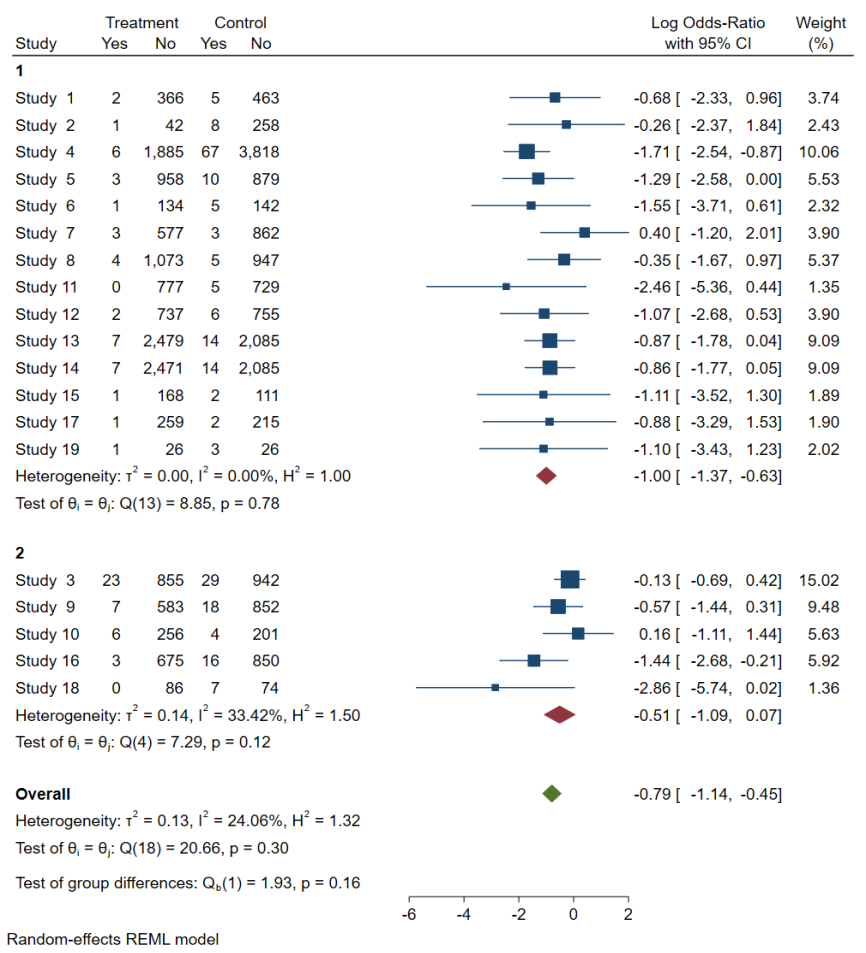
**

**Figure 27 Forest plot for the dichotomized age subgroup REML sensitivity analysis**

**
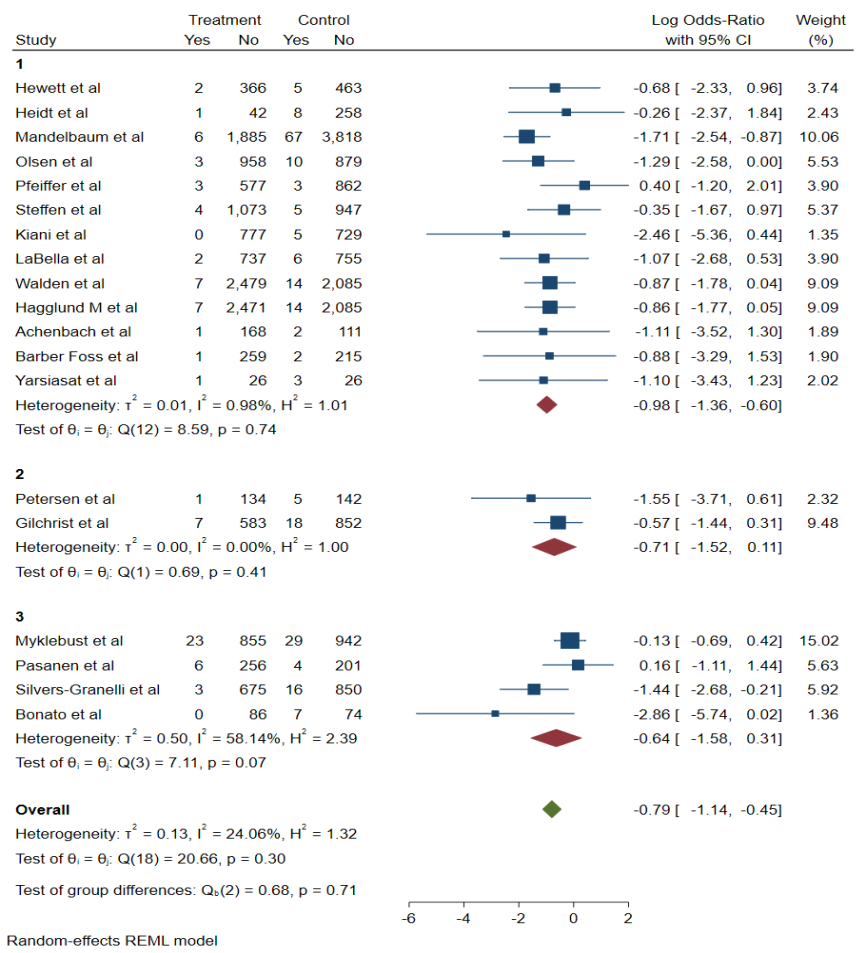
**

**Figure 28 Forest plot for the three-category age subgroup**

****3.2.2 Female sample****

****3.2.2.1** Data results**

****
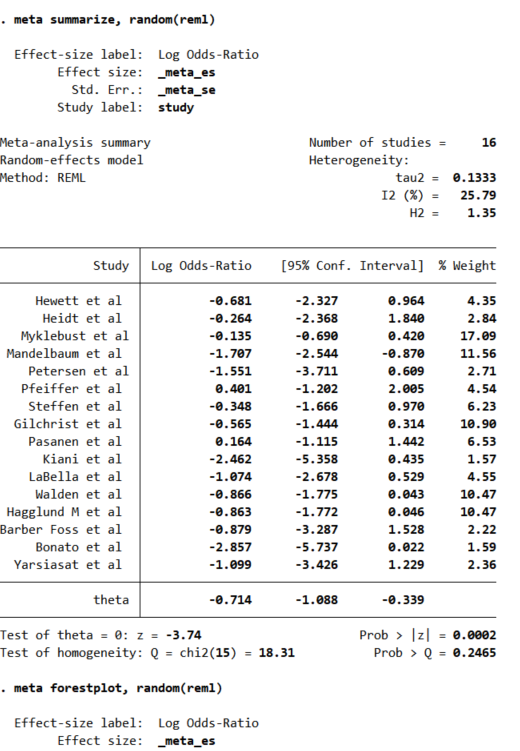
****

**Figure 29 REML sensitivity analysis results**


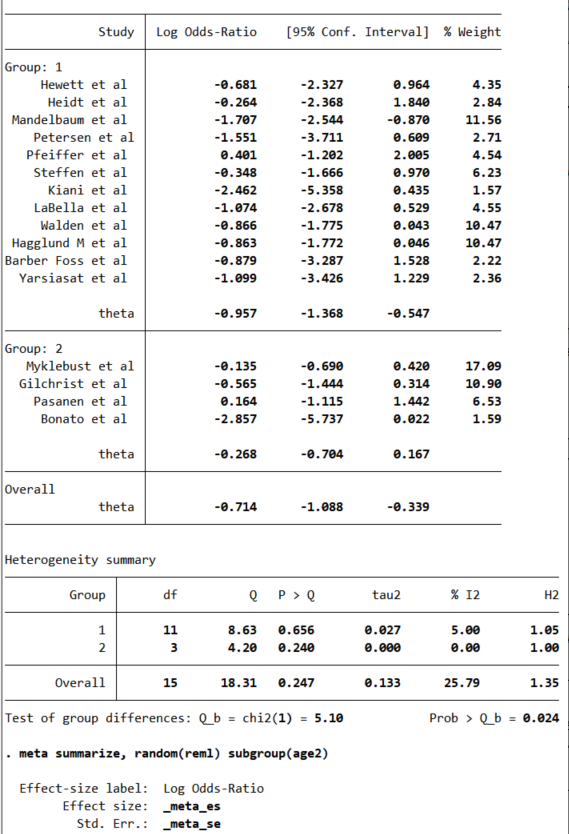


**Figure 30 REML sensitivity analysis results for the dichotomized age subgroup analysis**


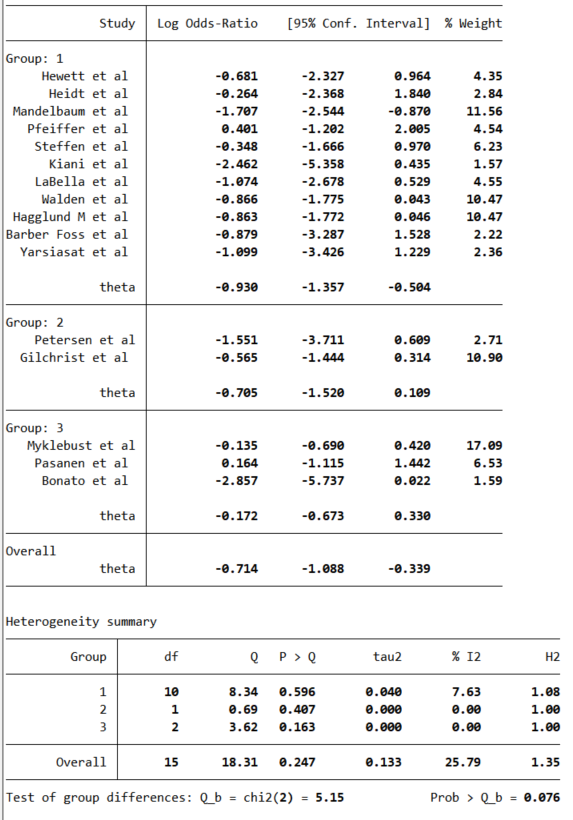


**Figure 31 REML sensitivity analysis results for the three-category age subgroup analysis**

**3.2.2.2 Forest plots**


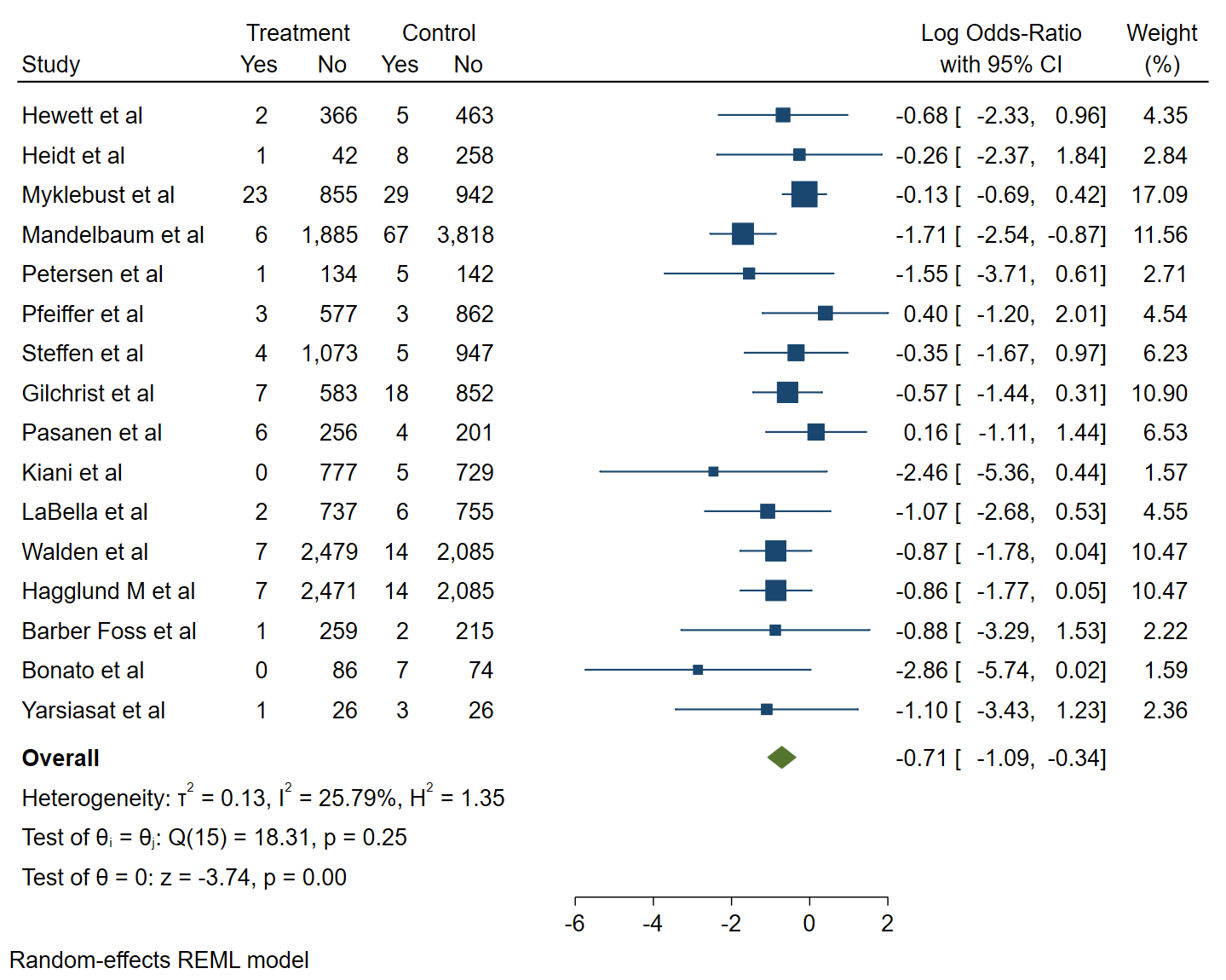


**Figure 32 Forest plot for the REML sensitivity analysis**

**
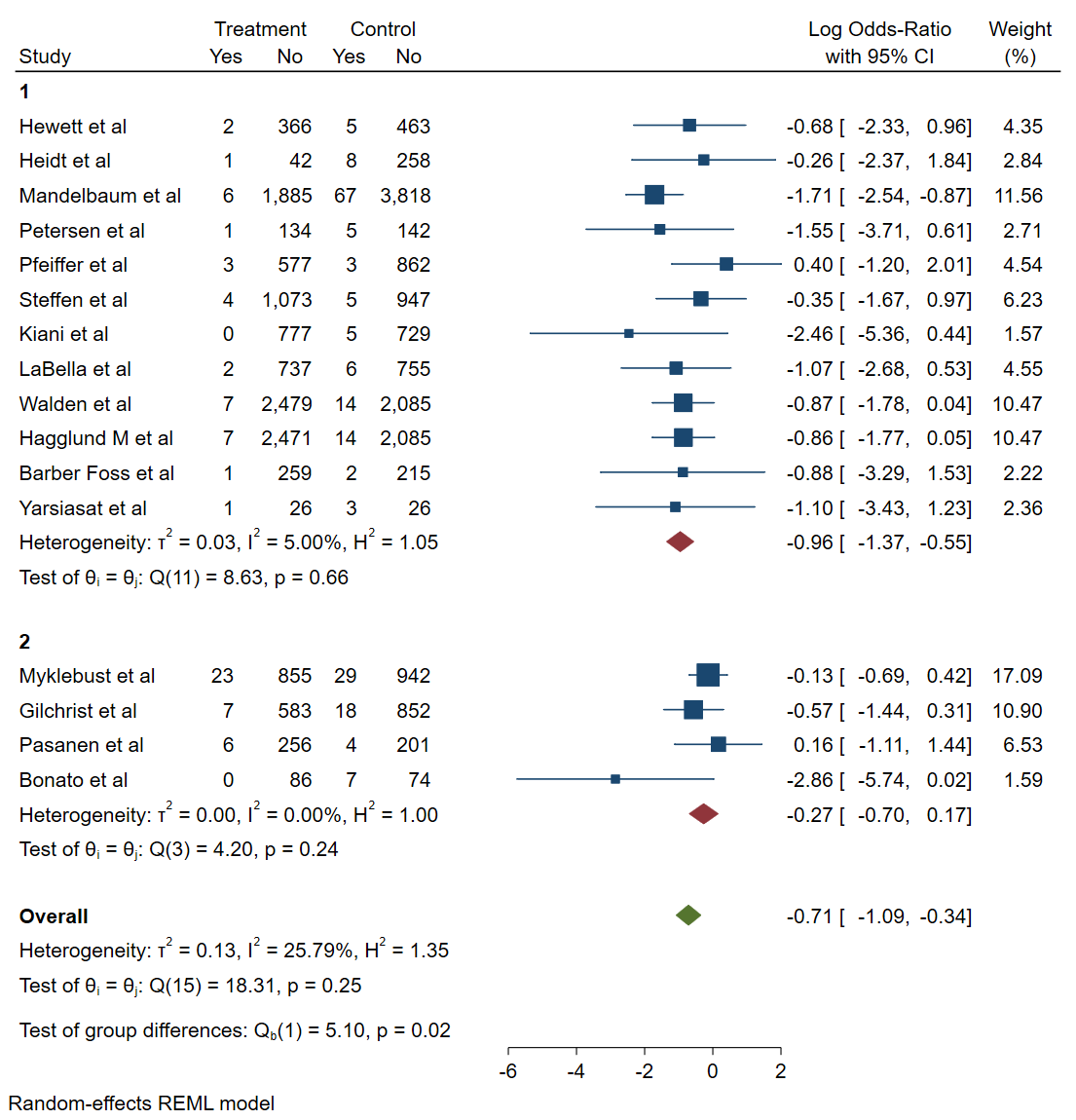
**

**Figure 33 Forest plot for the dichotomized age subgroup REML sensitivity analysis**

**
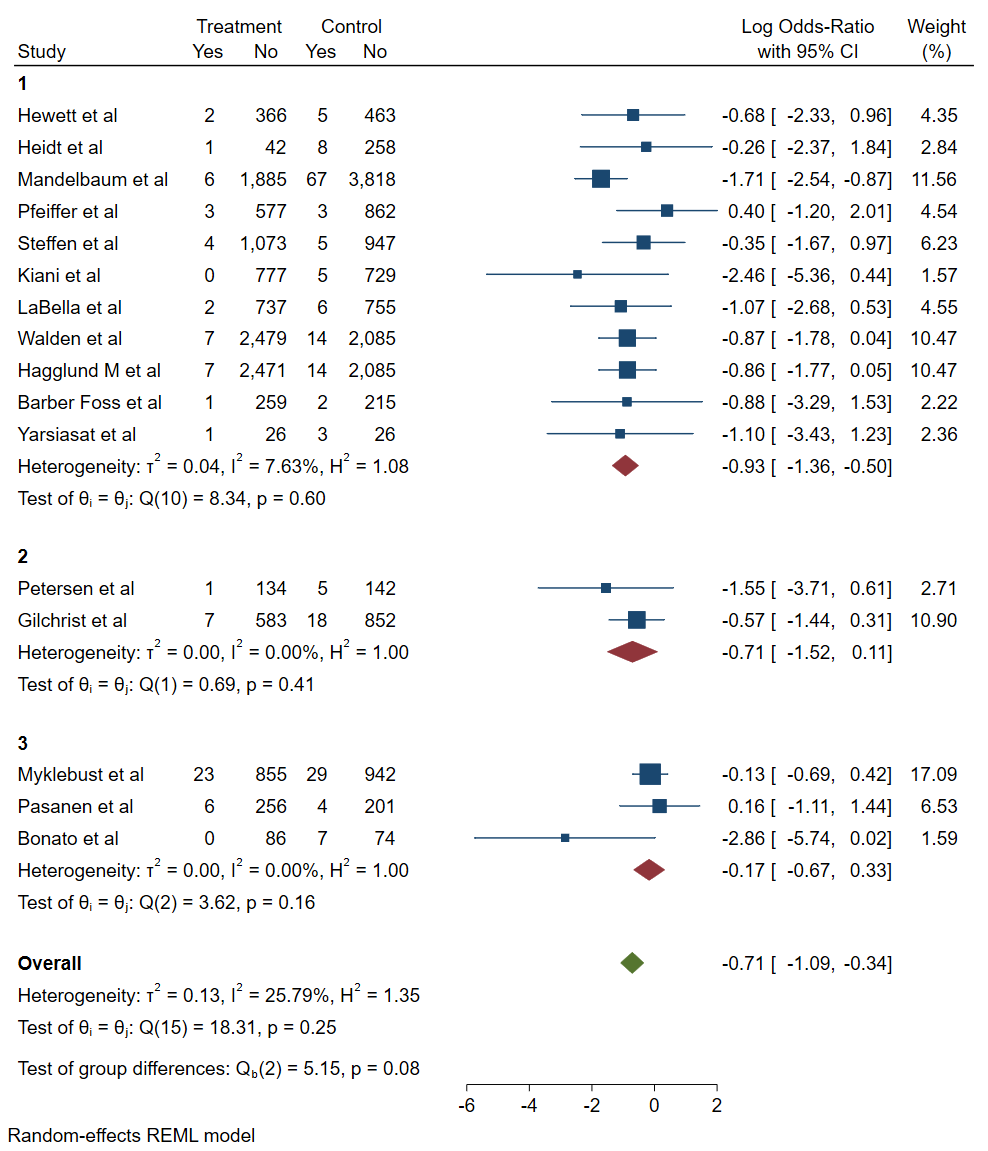
**

**Figure 34 Forest plot for the three-category age subgroup REML sensitivity analysis**

## ****4 Publication bias****

### ****4.1 Overall sample****

**
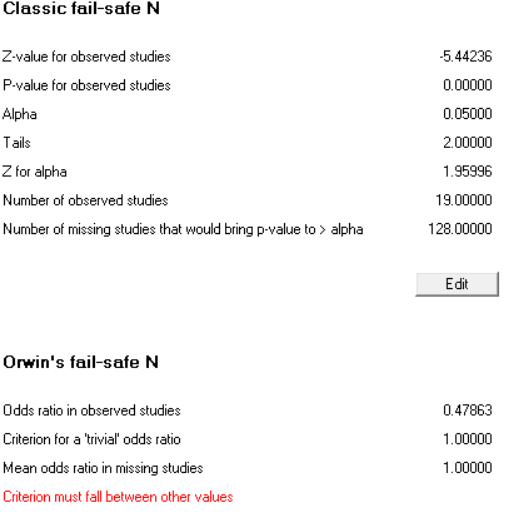
**

Figure 35 Classic fail-safe N for the overall sample

**
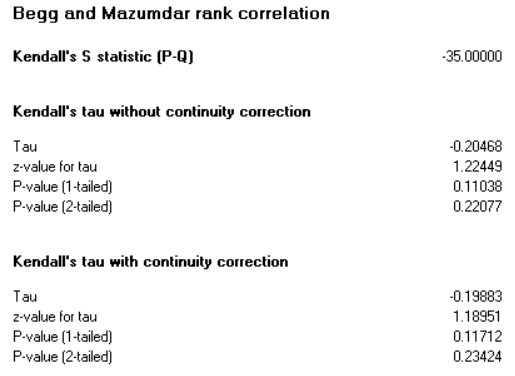
**

Figure 36 Begg–Mazumdar test for the overall sample

**
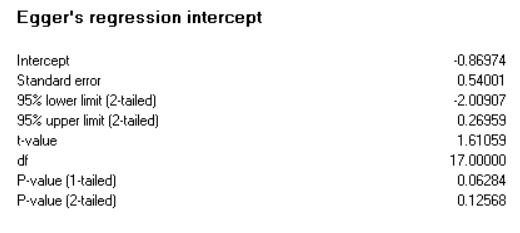
**

Figure 37 Egger’s test for the overall sample

**
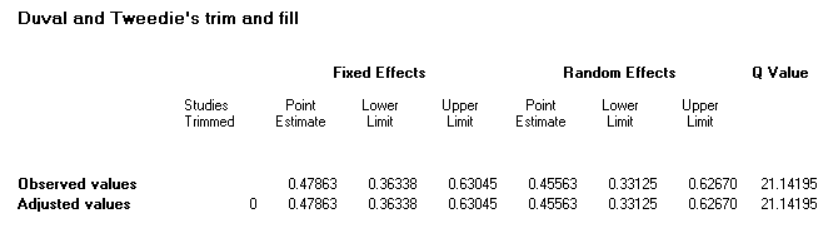
**

Figure 38 Trim-and-fill analysis for the overall sample

Figure 39 Funnel plot for the overall sample

**4.2 Female sample**


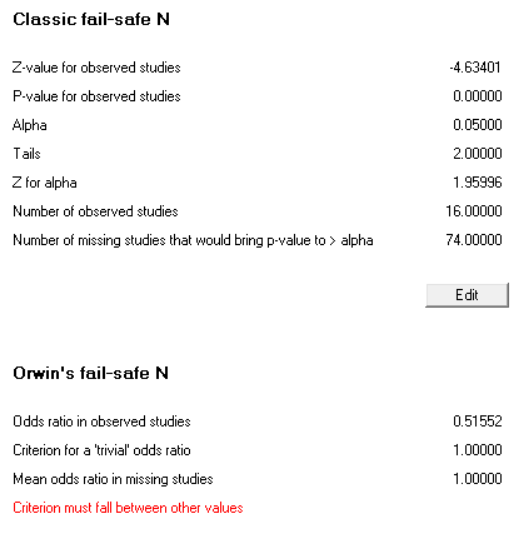


Figure 40 Classic fail-safe N for the female sample


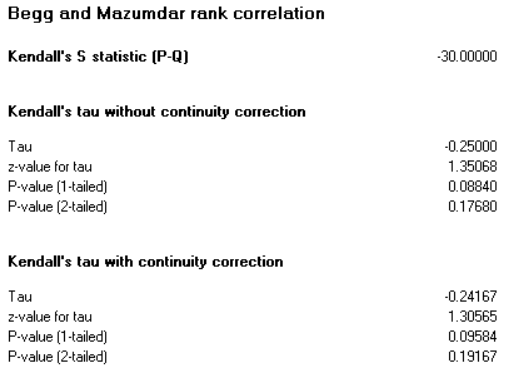


Figure 41 Begg–Mazumdar test for the female sample


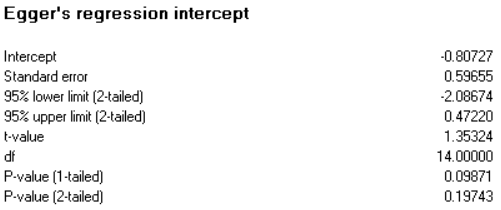


Figure 42 Egger’s test for the female sample


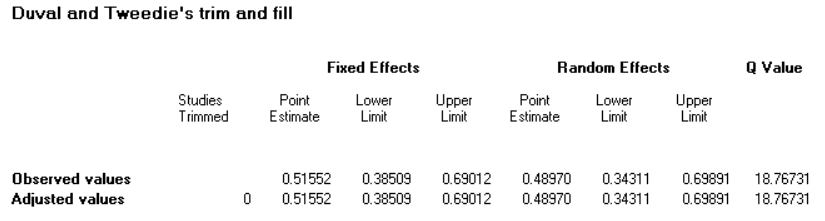


Figure 43 Trim-and-fill analysis for the female sample

Figure 44 Funnel plot for the female sample
